# Diversity-Oriented Synthesis and Optical Properties of Bichromophoric Pyrrole-Fluorophore Conjugates

**DOI:** 10.3389/fchem.2018.00579

**Published:** 2018-11-27

**Authors:** Oliver Grotkopp, Bernhard Mayer, Thomas J. J. Müller

**Affiliations:** Institut für Organische Chemie und Makromolekulare Chemie, Heinrich-Heine-Universität Düsseldorf Düsseldorf, Germany

**Keywords:** absorption, bichromophores, DFT, emission, energy transfer, level-2 functionalization, multicomponent reaction, pyrrole

## Abstract

The mild reaction conditions of the palladium-copper coupling-isomerization reaction open a highly convergent, chromogenic route to blue emissive pyrroles in the sense of a consecutive four-component reaction. By virtue of this strategy a phenol derivative can be readily accessed, which can be transformed in a level-2 transformation to a library of bichromophoric pyrrol-fluorophore conjugates by facile alkylation with fluorophore halides. The photophysics of the underlying blue emitter derivative and the conjugates is studied by absorption and emission spectroscopy, furnishing intramolecular energy transfer at short distances as well as competing fluorescence quenching. In some cases partial energy transfer results in the occurrence of dual emission, for instance seen as magenta-rose emission arising from blue and red orange luminescence. The experimental photophysical studies are rationalized by DFT and TD-DFT calculations.

## Introduction

A particularly interesting aspect of functional organic materials (Müller and Bunz, 2007) is based on inter- and intra-molecular interactions of chromophores, eventually, as multichromophore systems (Bazan, 2007). Non-conjugatively ligated multichromophores will not interact in the electronic ground state, if rigidified orientations and intramolecular aggregation are excluded, but their interaction occurs after photonic excitation, i.e., in the electronically excited states. Luminescence as an excited state phenomenon is particularly intriguing because it bears an enormous potential of application, ranging from fundamental science in molecular photonics (for reviews, see e.g., Fox, 1999; Garnier, 1999; Tour, 2000; Carroll and Gorman, 2002; Coropceanu et al., 2007; Shirota and Kageyama, 2007; Walzer et al., 2007) to illumination technology by OLED (organic light emitting diodes) (for reviews, see e.g., Müllen and Scherf, 2006; Park et al., 2011; Thejo Kalayani and Dhoble, 2012; Li, 2015). For modulation of emission colors not only is relevant for environment sensitive mapping of cellular compartments and structures (Klymchenko, 2017), but also in white light generation (Yuan et al., 2013) in the sense of additive color mixing. Most crucial in this context is avoidance of energy transfer cross-talk that proceeds within Förster radii. At usual concentration two chromophores emit independently in solution (Sarkar et al., 2016). Likewise this effect can also be achieved by embedding in micelles, organic or hybrid matrices (For recent examples of photochromic and multichromophoric emitters embedded in micelles or matrices, see e. g., Findlay et al., 2014; Shi et al., 2015; Bälter et al., 2016; Joshi et al., 2016; Börgardts and Müller, 2017; Pallavi et al., 2018). Far more challenging, however, is the conceptual design of unimolecular bi- or multichromophores capable of polychromic emission (for reviews on organic white light emitting devices and materials, see Mukherjee and Thilagar, 2014, 2015; Wu and Ma, 2016). In this case the occurrence of dual (or even polychromic) emission has to operate at intramolecular chromophore-chromophore distances around 1 nm, where excited state resonance energy transfer is ultrafast (for a detailed study on a umbelliferone–alizarin bichromophore, see Lapini et al., 2014). The control of energy transfer cross-talk of the constituting luminophores has to be modulated by partial and frustrated energy transfer (Klymchenko et al., 2003, 2007). While frustrated energy transfer unimolcular bichromophores operated by ESIPT (excited state intramolecular proton transfer) have been disclosed (for representative small molecule emitters operated by frustrated and partial energy transfer by ESIPT, see e.g., Park et al., 2009; Kwon et al., 2013; Benelhadj et al., 2014), we reasoned linear and angular geometrical orientation in emissive bichromophores might be achieved by level-2 functionalization of a blue emitter with several bathochromically emitting chromophores should lead to dually solution emissive unimolecular bichromophores.

**Graphical Abstract:**
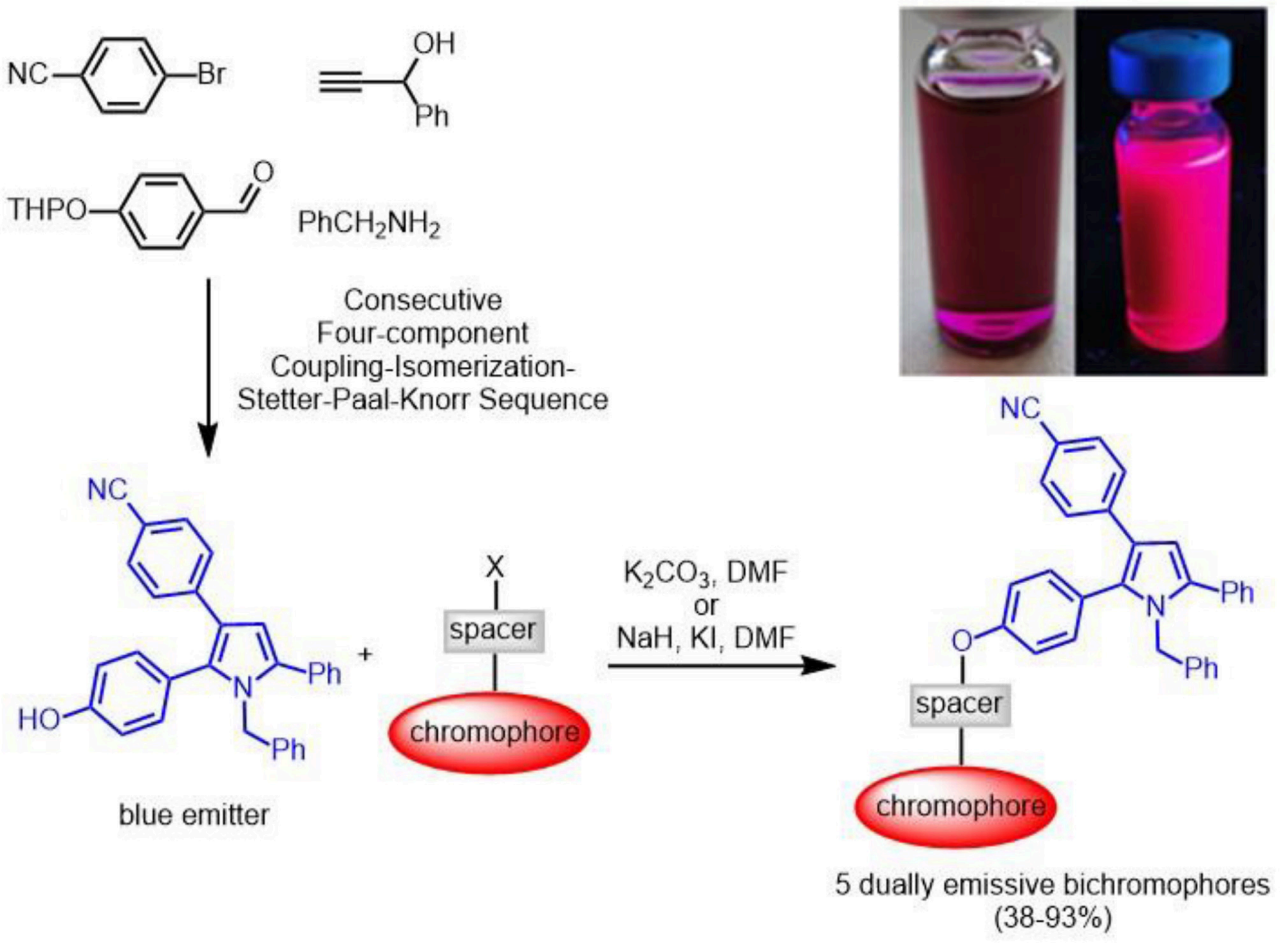
Concise two-step pyrrole-based non-conjugated emissive bichromophores by four-component pyrrole synthesis and nucleophilic substitution.

**Figure 1:**
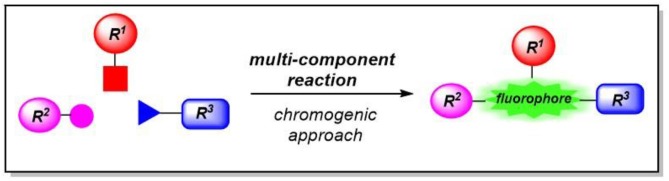
Diversity oriented syntheses of fluorophores by chromogenic multi-component reactions.

Diversity-oriented syntheses of dyes (for reviews on diversity-oriented syntheses of π-systems, see Briehn and Bäuerle, 2002; Müller, 2007; Müller and D'Souza, 2008; de Moliner et al., 2017) in a one-pot fashion have become an attractive tool for designing and optimizing chromophores. In this context, we have predominantly been focusing on developing chromogenic multicomponent syntheses of functional chromophores (Levi and Müller, 2016b), fluorophores (Figure 1) (Levi and Müller, 2016a; Riva et al., 2016), and aggregation-induced emissive chromophores (Müller, 2016; Merkt and Müller, 2018).

Several years ago we disclosed a consecutive four-component synthesis of blue-emissive pyrroles by a coupling-isomerization-Stetter-Paal-Knorr sequence (Braun et al., 2001; Braun and Müller, 2004). Herein, we report concise two step syntheses of a selection of bichromophoric pyrrole-fluorophore conjugates based upon the MCR pyrrole synthesis and its level two functionalization with a second redshifted emissive chromophore via Williamson ether synthesis or sulfonate formation. The electronic properties are conducted with absorption and fluorescence spectroscopy as well as interpreted in the light of DFT and TD DFT calculations.

## Results and discussion

### Synthesis

**Scheme 1:**
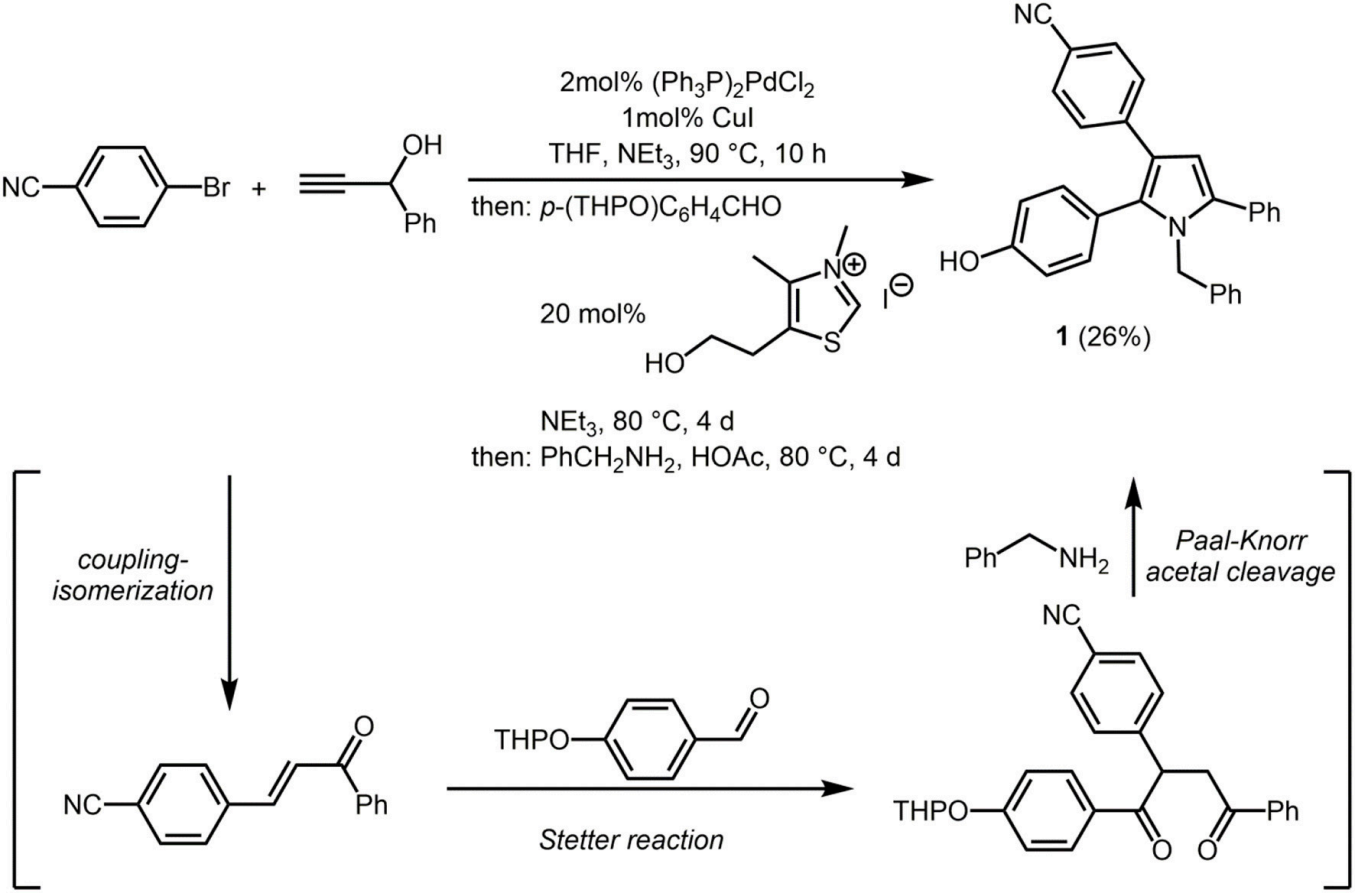
Consecutive four-component coupling-isomerization-Stetter-Paal-Knorr synthesis of 4-(1-benzyl-2-(4-hydroxy-phenyl)-5-phenyl-1*H*-pyrrol-3-yl)benzonitrile (**1**).

First attempts to employ the four-component coupling-isomerization-Stetter-Paal-Knorr pyrrole synthesis (Braun et al., 2001; Braun and Müller, 2004) to introduce the second chromophore in a one-pot fashion failed, predominantly due to solubility issues. Therefore, we envisioned that a phenol containing pyrrole might ideally serve for a post MCR ligation by etherification or esterification. Therefore, performing the four-component pyrrole synthesis with THP-protected *para*-hydroxybenzaldehyde gave rise to the formation of 4-(1-benzyl-2-(4-hydroxy-phenyl)-5-phenyl-1*H*-pyrrol-3-yl)benzonitrile (**1**) after isolation and chromatographic purification in 26% (Scheme 1). Under the acidic conditions of the terminal Paal-Knorr cyclocondensation the THP group is cleaved to give the free phenol.

Absorption spectroscopy of phenol **1** reveals a longest wavelength absorption maximum at 319 nm and an additional absorption band at 275 nm. Upon excitation at the longest wavelength maximum a broad intense emission at 442 nm is found with a relative fluorescence quantum yield Φ_F_ of 0.11, i.e., in a comparable magnitude as other benzonitrile substituted pyrroles (Braun and Müller, 2004) (vide infra). With this intensively blue emissive building block in hand the stage was set for the synthesis of luminescent bichromophores in the sense of a level-2 functionalization.

**Scheme 2:**
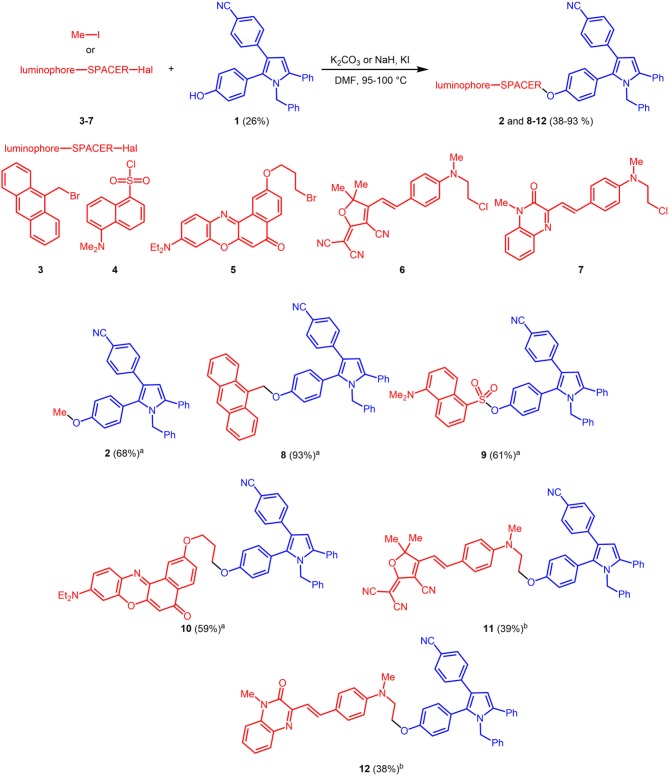
Synthesis of reference pyrrole **2** and fluorescent bichromophores **8**–**12** by base mediated etherification or sulfonylation of phenol **1** (blue emitter) and luminophore derivatives **3–7** (redshifted emitters) or methyl iodide (^a^K_2_CO_3_; ^b^NaH, KI).

The reference luminophore **2**, a methyl ether derivative, was synthesized by etherification of phenol **1** with methyl iodide in 68% yield. Halide functionalized luminophores **3** (Aathimanikandan et al., 2005) and **5** (Kucherak et al., 2010), **6** (Lord et al., 2010), and **7** (Lord et al., 2010) (some also containing alkyl spacers) were prepared according to literature protocols (dansyl chloride was purchased and used as received) and submitted to base mediated Williamson ether synthesis or sulfonylation with phenol **1** to give bichromophores **8**–**12** in moderate to excellent yield (Scheme 2). While the bromides **3** and **5**, dansyl chloride **4** and methyl iodide react smoothly with potassium carbonate as a base, for the chlorides **6** and **7**, Finkelstein conditions with potassium iodide have to applied, which work best in these cases with sodium hydride as a base.

The ^1^H and ^13^C NMR and mass spectra (MALDI-TOF and HRMS) unambiguously confirm the successful ligation of the **1** with the second chromophores **3–7** as well as methyl iodide, and thereby the structures of pyrrole reference chromophore **2** and the bichromophores **8–12**.

### Photophysical properties and electronic structure

Upon excitation with a handheld UV lamp the pyrrole reference chromophore **2** and all bichromophores **8–12** luminesce, indicating that the photonic excitation is neither completely quenched by internal conversion nor by electron transfer into long-lived charge separated states arising from photoinduced intramolecular electron transfer (PIET) (Kavarnos, 1993). This prompted us to study the absorption and emission spectra of the luminophores **2** and **8–12** in more detail (Table 1).

**Table 1:** Selected absorption and emission data of luminophores **2**, reference luminophores, and bichromophores **8–12** [recorded in dichloromethane at concentrations of 10^−5^ m (absorption) and 10^−7^ m (emission) at *T* = 293 K]^a^.

**Compounds**	**Absorption**	**Emission**	**Stokes shift^a^**	**Emission color^b^**
	***λ***_max, abs_ **[nm]**	***λ***_max, em_ **[nm]**	$\Delta \tilde{\nu }$ **[cm**^−1^**]**
	(ε) [L mol^−1^ cm^−1^]	(*Φ_*f*_*)	
**2**	276 (30,600), **316** (20,200)	**445.5** (0.28)^d^	9,200	blue
9-methyl anthracene (Roberts and Yavari, 1981)	332 (4,400), 348 (5,400), 365 (12,900), **385** (11,000)	**388**, 411 (0.36) (Rice et al., 1980)	200	blue
dansyl phenolate (Beyeh et al., 2007)^h^	263 (16,800), **350** (4,600)	**515**	9,200	Green
**8**	258 (192,800), 315.5 (29,800), 329 (29,600), 367 (18,100), **387** (14,200)	**394**, 415.5, 439.5 (0.09)^c^, ^d^ **443.5**^d^, ^e^	500 9,000	Blue
**9**	266 (54,400), **318** (32,200)	**519** (0.28)^f^ 444 (sh)	12,700	Green
**10**	267.5 (16,200), 294 (7,200), 316 (6,000), **529.5** (10,500)	**596**	2,000	Red
**11**	302 (34,700), **566** (51,900)	442, **618**^g^	1,500	Purple
**12**	267 (54,700), **446** (51,700)	445 (< 0.01)^d^, **551** (0.04)^f^	4,200	Yellow orange

a *The absorption maxima employed for calculating the Stokes shifts ($\Delta \tilde{\nu }$ = 1/λ_max, abs_ – 1/λ_max, em_ [cm^−1^]) are marked bold face*.

b *Emission color upon excitation with a handheld UV lamp (λ_exc_ = 365 nm)*.

c *Fluorescence upon excitation at λ_exc_ = 260, 367, and 387 nm*.

d *Determined with 1,9-diphenylanthracene as a standard (cyclohexane, Φ_f_ = 1.00)*.

e *Fluorescence upon excitation at λ_exc_ = 317 and 330 nm*.

f *Determined with dansyl glycine as a standard (1,4-dioxane, Φ_F_ = 0.66)*.

g *Upon excitation at λ_exc_ = 365 nm*.

h *Recorded in chloroform*.

The absorption spectra of all bichromophores **8–12** behave essentially additively with respect to the underlying subchromophores. This is quantitatively demonstrated by comparison of the UV/Vis spectrum of the pyrrole-anthracene bichromophore **8** and the sum spectra of the reference chromophores **2** (pyrrole) and 9-methyl anthracene (anthracene) (for spectral details, see Supplementary Material). As expected, in the electronic ground state, from where photonic excitation starts, the two subchromophores essentially neither interact nor form aggregates at the concentrations investigated. However, in the excited state, as studied by fluorescence spectroscopy, in some cases a cooperative behavior exists, which is also dependent on the excited subchromophore.

**Figure 2:**
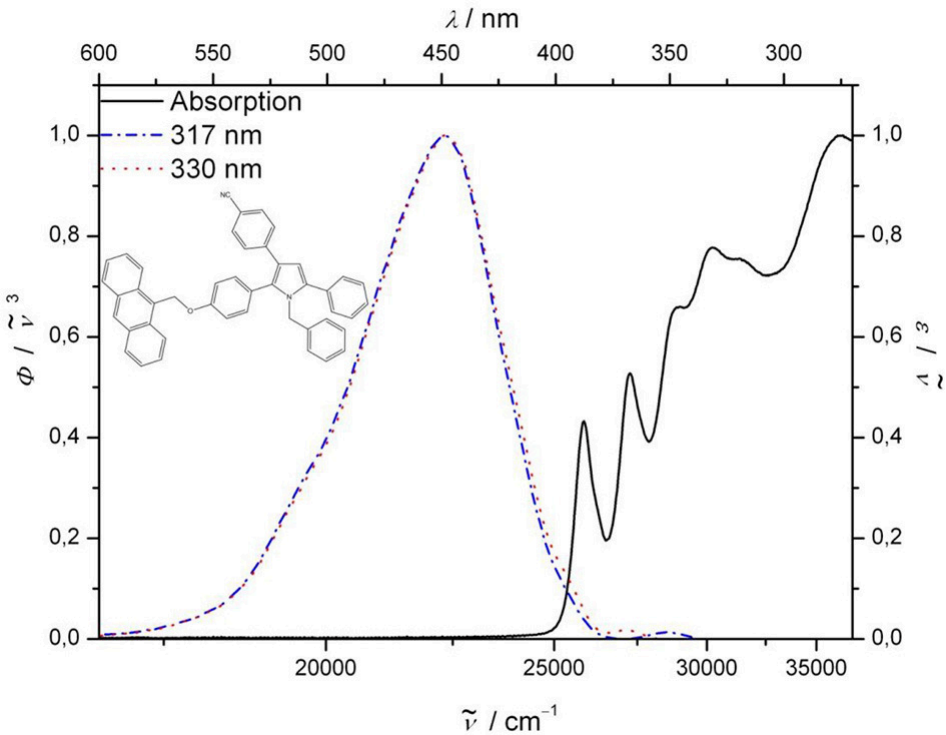
Normalized UV/Vis (solid lines) and emission spectra (dotted: λ_*exc*_ = 330 nm; dash-dotted: λ_*exc*_ = 317 nm) of bichromophore **8** (recorded in CH_2_Cl_2_ at *T* = 298 K).

Upon excitation of the pyrrole-anthracene bichromophore **8** at the absorption bands of the anthracene chromophore at 260, 367, and 387 nm clearly the emission bands of anthracene with vibrational resolution are detected (for spectral details, see Supplementary Material). However, upon excitation at the pyrrole absorption bands at 317 and 330 nm exclusively the pyrrole typical structureless emission band at 443.5 nm appears (Figure 2). The separate excitations of the discrete absorption bands of the subchromophores in bichromophore **8** are not accompanied by energy transfer from the donor (pyrrole) to the acceptor (anthracene). Although the fluorescence quantum yield Φ_*f*_ of 0.09 accounts for a significant energy dissipation upon excitation of the anthracene moiety, the lack of overlap of the absorption band of the acceptor with the emission band of the donor and, hence, the absence of energy transfer suggests that in bichromophore **8** are not electronically coupled.

In the other bichromophores **9**–**12** bearing redshifted absorptions of the acceptor chromophores the situation of the emission characteristics changes (for spectral details, see Supplementary Material).

The absorption bands of the dansyl-pyrrole (**9**), the Nile red-pyrrole (**10**), and the quinoxalinyl-styryl-pyrrole (**12**) bichromophores clearly possess significant energy transfer characteristics, where the selective excitation of the pyrrole donor chromophore with its emission band more (**10**, **12**) or less (**9**) overlaps with the absorption bands of the corresponding acceptor chromophores. The efficiency of the energy transfer Φ_*EnT*_ can be estimated according to the formula (Qing et al., 2011) $\Phi_{EnT}=1-\;\frac{\Phi_{Donor}}{\Phi_{Donor}^{\text{0}}}$, where Φ_*donor*_ is the measured quantum yield of the (residual) donor emission band of the bichromophore and $\Phi_{donor}^{0}$ is the quantum yield of the donor chromophore. Determination of Φ_*Donor*_ of the residual pyrrole emission at 445 nm furnishes for bichromophore **12** a quantum yield of the energy transfer Φ_*EnT*_ of over 0.97. For the dansyl bichromophore **9** Φ_*EnT*_ can be estimated to be >0.98, while for the Nile red bichromophore **10** no residual emission around 450 nm can be detected, indicating an Φ_*EnT*_ of unity. However, it has to be kept in mind that an exergonic photoinduced intramolecular electron transfer (PIET) cannot be fully excluded, in particular, since the determined fluorescence quantum yields are relatively low. For the anthryl-pyrrole bichromophore **8** the PIET can be estimated to be endothermic.

**Figure 3:**
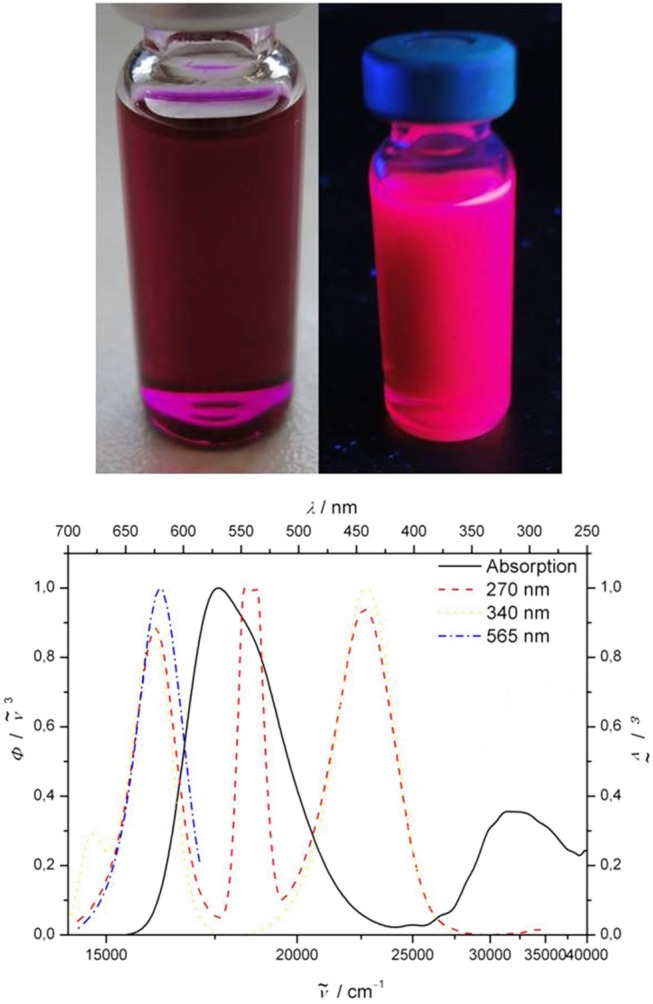
**(Top)** Dichloromethane solution of bichromophore **11** at day light and under the handheld UV lamp (λ_*exc*_ = 365 nm). **(Bottom)** Normalized absorption (black solid line) and emission spectra at λ_*exc*_ = 565 (blue dashed), 340 (yellow dashed), and 270 nm (red dashed) (recorded in dichloromethane at *T* = 298 K; the intense, second order signal at 540 nm upon excitation at λ_*exc*_ = 270 nm is its first harmonic).

Most remarkable, however, are the emission characteristics of the 3-cyano-5,5-dimethylfuran-2(5*H*)-ylidene)malononitrile-styryl bichromophore **11**, where a strong dependence on the excitation wavelength λ_*exc*_ can be detected. The dichloromethane solution of bichromophore **11** does not show the typical red emission of the acceptor chromophore upon eyesight at excitation with 254 or 365 nm by a handheld UV lamp, but rather a magenta-rose emission with significant intensity (Figure 3, top). This mixing emission color between violet (λ_*max, em*_ = 442 nm) and orange red (λ_*max, em*_ = 618 nm) almost matches with the line of purples (Westland, 2003; Broadbent, 2004; Schanda, 2007). The emission spectra at various excitation wavelength clearly reveal that an excitation at 365 nm not only causes an energy transfer from the pyrrole donor to the 3-cyano-5,5-dimethylfuran-2(5*H*)-ylidene)malononitrile-styryl acceptor (Figure 3, bottom), which emits at 618 nm, but also an emission of the donor itself at 442 nm. This peculiar behavior can be interpreted in the sense of a partial energy transfer (for representative small molecule emitters operated by frustrated and partial energy transfer by ESIPT, see e.g., Park et al., 2009; Kwon et al., 2013; Benelhadj et al., 2014), i.e., a dual emission as a consequence of an excited state communication between donor and acceptor.

For a deeper understanding of the observed chromophore-chromophore interactions TD-DFT calculations were performed on the pyrrole **2** and the bichromophores **8–12**. The geometries of the electronic ground-state and excited structures were optimized by using Gaussian09 (Frisch et al., 2009) with the B3LYP functional (Lee et al., 1988; Becke, 1993; Kim and Jordan, 1994; Stephens et al., 1994) and the Pople 6-311G^**^ basis set (Krishnan et al., 1980). Since absorption properties were measured in dichloromethane solutions, the polarizable continuum model (PCM) with dichloromethane as a solvent was utilized (Scalmani and Frisch, 2010). All minimum structures were unambiguously assigned by analytical frequency analysis. The optimized structures were then submitted to TD-DFT calculations employing the gradient-corrected exchange and correlation Perdew-Burke-Ernzerhof functionals PBE_1_PBE (Adamo and Barone, 1999)/6-311^**^ (Krishnan et al., 1980) with dichloromethane (IEFPCM) (Scalmani and Frisch, 2010) as a solvent.

**Figure 4:**
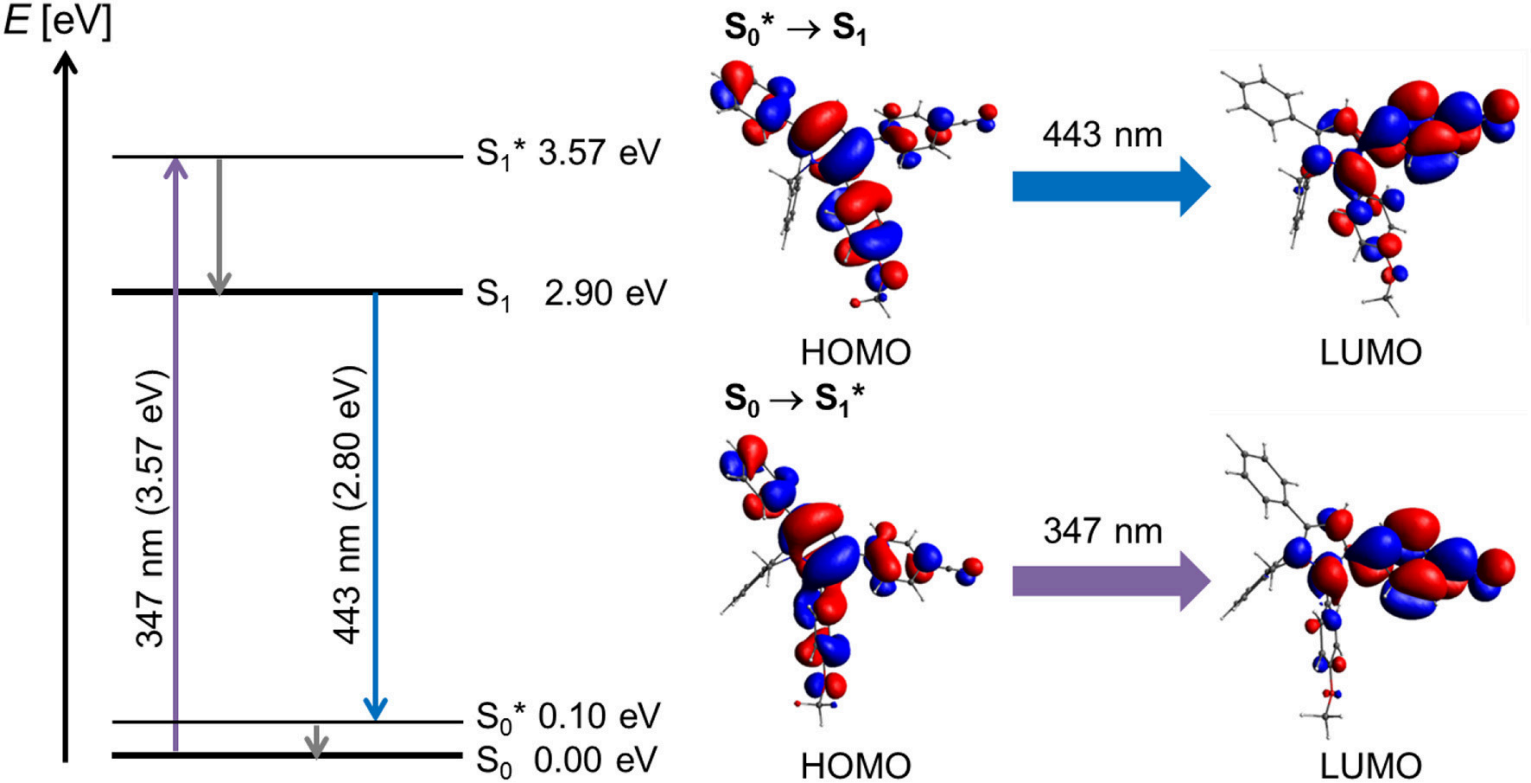
Jablonski diagram of compound **2** and assignment of the FMO-transitions in the longest wavelength absorption band and the emission band [*E*(S_0_) = 0 eV; PBE_1_PBE 6-311G** IEFPCM CH_2_Cl_2_, isosurface value at 0.03 a.u.].

The blue emissive pyrrole **2** was considered as the model donor chromophore in the bichromophore systems. The TD-DFT calculation of structure **2** revealed, in reasonably good agreement with the experimentally determined longest wavelength absorption band at 316 nm (for detailed calculated transitions, see Table S1), a lowest energy transition at 347 nm for the S_1_ Franck-Condon absorption, which is represented to 99% as a HOMO → LUMO transition with considerable charge transfer character from the anisyl and phenyl moieties of the pyrrole to the *p*-cyanophenyl acceptor (Figure 4). The excitation from the vibrationally excited ground state S${}_{0}^{*}$ to the relaxed first excited state S_1_ translates to the process of fluorescence. The involved HOMO → LUMO transition (99%, *f* = 0.2298, λ_*calc*_ = 443 nm, λ_*max, exp*_ = 446 nm) almost exactly reverts the electron coefficient density distribution of the absorption.

For further discussion only the absorption characteristics of the bichromophores **8**-**12** were considered (for Jablonski diagrams of the dominant absorption bands, see Figures S12–S16). The calculations support the additive nature of the absorption bands of the constituting chromophores, i.e., all chromophore-chromophore interactions occur in the excited state. As observed experimentally, energy transfer as an envisioned interaction requires sufficient overlap of the emission band of the pyrrole and the absorption band of the corresponding ligated second chromophore. If a dual emission is intended as observed for bichromophore **11**, for creating dual emission color mixing, it becomes obvious that partial energy transfer is more favorable than complete Förster resonance energy transfer.

## Conclusion

The consecutive four-component coupling-isomerization-Stetter-Paal-Knorr synthesis of blue luminescent pyrrole has been employed to furnish a pyrrole chromophore, which can be successfully ligated in the sense of a level-2 functionalization with emitter chromophores, absorbing at longer wavelengths to give a library of emissive bichromophores. The photophysical data could be quickly assessed by absorption and emission spectroscopy and were rationalized by TD-DFT calculations. While significant overlap of the absorption bands of both chromophores do not reveal a peculiar interaction in the excited state, those bichromophores where the second absorption bands overlap with the pyrrole emission reveal energy transfer characteristics. In the case of the reddish purple chromophore dual emission from the pyrrole and the 3-cyano-5,5-dimethylfuran-2(5*H*)-ylidene)malononitrile-styryl moiety clearly results from partial energy transfer causing a magenta-rose emission with significant intensity. This diversity-oriented synthetic principle now enables a rapid synthetic approach to dually emissive unimolecular bichromophores operating by partial energy transfer. The novel principle can be envisioned to be employed for accessing unimolecular white light emitters for OLED and biophysical analytics. Synthetic and photophysical studies of similar blue-red emitting bichromophores are currently underway.

## Experimental

### 4-(1-benzyl-2-(4-hydroxyphenyl)-5-phenyl-1*H*-pyrrol-3-yl)benzonitrile (1)

In a screw-cap Schlenk vessel with a magnetic stir bar were placed dry THF (10mL), *p*-bromo benzonitrile (910mg, 5.00 mmol), 1-phenylprop-2-yn-1-ol (660 mg, 5.00 mmol), PdCl_2_(PPh_3_)_2_ (70mg, 0.10 mmol), CuI (40mg, 0.20 mmol), and triethylamine (1.7mL, 12 mmol) under nitrogen and the mixture was stirred at 90°C (oil bath) for 10 h. Then, after cooling to room temp, 4-(tetrahydro-2*H*-pyran-2-yloxy)benzaldehyde (1.10 g, 5.25mmol), 3,4-dimethyl-5-(2-hydroxyethyl)-thiazolium iodide (385mg, 1.35 mmol), and triethylamine (2.5mL, 18mmol) were added and the mixture was stirred at 80°C for 4 d. After cooling to room temp acetic acid (10mL) and benzyl amine (2.6 g, 25mmol) were added and the reaction mixture was stirred at 80°C for 3 d. After cooling to room temp a saturated aqueous sodium carbonate solution was added, the phases were separated and the aqueous phase was extracted with diethyl ether (3 × 50mL). The combined organic layers were dried (anhydrous sodium sulfate), filtered and the filtrate was adsorbed on celite^®;^ and purified by flash chromatography on silica gel (hexane/ethyl acetate 20:1) to give pyrrole **1** as a colorless solid (554mg, 26%), Mp 202°C.

^1^H NMR (500 MHz, CDCl_3_): δ 5.15 (s, 2 H), 6.7 (m, 3 H), 6.82 (d, ^3^*J* = 8.6 Hz, 2 H), 7.07 (d, ^3^*J* = 8.6 Hz, 2 H), 7.12 (m, 2 H), 7.29 (m, 1 H), 7.36 (m, 3 H), 7.41 (m, 2 H), 7.46 (m, 2 H), 7.52 (m, 2 H), 8.61 (s, 1 H). ^13^C NMR (126 MHz, CDCl_3_): δ 49.4 (CH_2_), 108.7 (C_quat_), 109.8 (CH), 116.5 (C_quat_), 116.6 (CH), 119.8 (C_quat_), 121.9 (C_quat_), 124.3 (C_quat_), 126.7 (CH), 127.7 (CH), 128.2 (CH), 128.3 (CH), 129.1 (CH), 129.4 (CH), 129.7 (CH), 132.7 (CH), 133.3 (CH), 134.2 (C_quat_), 134.9 (C_quat_), 136.6 (C_quat_), 139.9 (C_quat_), 142.5 (C_quat_). MALDI-TOF [*m/z* (%)]: 426.0 ([M]^+^, 100). IR (KBr): $\tilde{\nu }$ [cm^−1^] = 3,342 (w), 2,229 (m), 1,601 (s), 1,516 (m), 1,450 (m), 1,402 (w), 1,338 (m), 1,267 (s), 1,228 (m), 1,172 (m), 1,072 (w), 939 (w), 839 (s). Anal. calcd. for C_30_H_22_N_2_O + H_2_O (426.5 + 18.01): C 81.06, H 5.44, N 6.30; Found: C 81.01, H 5.01, N 6.14. HRMS (ESI) calcd. for [C_30_H_22_N_2_O – H]^+^: 425.16484; Found: 425.16485.

### General procedure (GP) for the synthesis of the pyrrole reference chromophore 2 and bichromophores 8–12

Variation A: Pyrrole **1** (1.00 equiv), halide **2–4** or methyl iodide (1.00 equiv), K_2_CO_3_ (2.00 equivs) and dry DMF (5 mL) were placed in a screw-cap Schlenk vessel with a magnetic stir bar under nitrogen (for details, see Table 2). The reaction mixture was heated at 100°C for 5 h. After cooling to room temp celite^®;^ was added to the reaction mixture and the solvents were removed in vacuo. The residue was purified by flash chromatography on silica gel (hexane/ethyl acetate) to give the pyrrole reference chromophore **2** or bichromophores **8–10**.

**Table 2:** Alkylation synthesis of pyrrole **2** and bichromophores **8–12**.

**Entry**	**Pyrrole 1**	**Alkylation substrate**	**Pyrrole 2 and bichromophores 8–12**
1^a^	43 mg (0.1 mmol)	50 mg (0.4 mmol) of methyl iodide	39 mg (68%) of **2**
2^a^	58 mg (0.1 mmol)	27 mg (0.1 mmol) of **3**	57 mg (93%) of **8**
3^a^	44 mg (0.1 mmol)	28 mg (0.1 mmol) of **4**	42 mg (61%) of **9**
4^a^	44 mg (0.1 mmol)	42 mg (0.1 mmol) of **5**	46 mg (59%) of **10**
5^b^	105 mg (0.25 mmol)	96 mg (0.25 mmol) of **6**	70 mg (39%) of **11**
6^b^	105 mg (0.25 mmol)	84 mg (0.25 mmol) of **7**	78 mg (38%) of **12**

a *According to variation A*.

b *According to variation B*.

Variation B: Pyrrole **1** (1.00 equiv), KI (1.00 equiv), and dry DMF (6 mL) were placed in a screw-cap Schlenk vessel with a magnetic stir bar under nitrogen. The mixture was cooled to 0°C (ice/water bath) and sodium hydride (2.00 equivs) was added. Then, halide **5** or **6** (1.00 equiv) was added and the mixture was allowed to come to room temp. Then, the mixture was stirred at 90°C for 3.5 h (for details, see Table 2). After cooling to room temp water (2 mL) was carefully added and the mixture was extracted with ethyl acetate (3 × 25 mL). The combined organic phases were dried (anhydrous sodium sulfate) and the solvents were removed in vacuo. The residue was purified by flash chromatography on silica gel (hexane/ethyl acetate 4:1) to give the bichromophores **11** or **12**.

#### 4-(1-benzyl-2-(4-methoxyphenyl)-5-phenyl-1H-pyrrol-3-yl)benzonitrile (2)

According to the GP (variation A) compound **2** (30mg, 68%) was obtained as a colorless solid, Mp 166°C.

^1^H NMR (300 MHz, CDCl_3_): δ 3.79 (s, 3 H), 5.05 (s, 2 H), 6.57 (s, 1 H), 6.66 (m, 2 H), 6.81 (d, ^3^*J* = 8.84 Hz, 2 H), 7.07 (d, ^3^*J* = 8.43 Hz, 2 H), 7.12 (m, 2 H), 7.24-7.42 (m, 10 H). ^13^C NMR (75 MHz, CDCl_3_): δ 48.6 (CH_2_), 55.4 (CH_3_), 108.1 (C_quat_), 109.0 (CH), 114.4 (CH), 119.8 (C_quat_), 121.5 (C_quat_), 124.7 (C_quat_), 126.2 (CH), 127.1 (CH), 127.6 (CH), 127.7 (CH), 128.5 (CH), 128.7 (CH), 129.3 (CH), 132.2 (CH), 132.5 (CH), 133.2 (C_quat_), 133.6 (C_quat_), 136.1 (C_quat_), 139.0 (C_quat_), 141.4 (C_quat_), 159.8 (C_quat_). MALDI-TOF [*m/z* (%)]: 440.05 ([M]^+^, 100). IR (KBr): $\tilde{\nu }$ [cm^−1^] = 2,224 (m), 1,601 (s), 1,576 (m), 1,516 (m), 1,489 (m), 1,470 (w), 1,386 (w), 1,354 (m), 1,306 (w), 1,290 (m), 1,253 (s), 1,178 (m), 1,109 (w), 1,028 (m), 1,016 (w), 839 (s), 769 (m), 759 (s), 723 (s), 698 (s). HRMS (ESI) calcd. for [C_31_H_24_N_2_O + H]^+^: 441.19614; Found: 441.19728.

#### 4-(2-(4-(anthracen-9-ylmethoxy)phenyl)-1-benzyl-5-phenyl-1H-pyrrol-3-yl)benzonitrile (8)

According to the GP (variation A) compound **8** (57 mg, 93%) was obtained as a colorless solid, Mp 202°C.

^1^H NMR (500 MHz, CDCl_3_): δ 5.10 (s, 2 H), 5.95 (s, 2 H), 6.58 (s, 1 H), 6.71 (d, ^3^*J* = 6.3 Hz, 2 H), 7.05 (m, 2 H), 7.15 (m, 5 H), 7.31 (m, 4 H), 7.38 (m, 2 H), 7.48 (m, 5 H), 7.55 (m, 2 H), 8.05 (d, ^3^*J* = 8.3 Hz, 2 H), 8.28 (d, ^3^*J* = 8.9 Hz, 2 H), 8.53 (s, 1 H). ^13^C NMR (126 MHz, CDCl_3_): δ 48.7 (CH_2_), 63.1 (CH_2_), 108.2 (C_quat_), 109.2 (CH), 115.5 (CH), 115.5 (CH), 119.8 (C_quat_), 121.6 (C_quat_), 124.1 (CH), 125.3 (CH) 125.3 (C_quat_), 126.2 (CH), 126.8 (C_quat_), 126.9 (CH), 127.2 (CH), 127.7 (CH), 128.6 (CH), 128.7 (CH), 129.3 (CH), 129.4 (CH), 129.4 (CH), 131.3 (C_quat_), 131.7 (C_quat_), 132.2 (CH), 132.7 (CH), 133.2 (C_quat_), 133.6 (C_quat_), 136.3 (C_quat_), 139.0 (C_quat_), 141.5 (C_quat_), 159.4 (C_quat_). MALDI-TOF [*m/z* (%)]: 617.2 ([M+H]^+^, 60). IR (KBr): $\tilde{\nu }$ [cm^−1^] = 2,224 (m), 1,603 (m), 1,516 (w), 1,489 (w), 1,450 (w), 1,244 (s), 1,224 (w), 1,175 (m), 1,001 (w), 989 (w), 835 (s), 762 (s), 731 (s), 698 (s). UV/Vis: λ_max_(ε) (CH_2_Cl_2_, *T* = 293 K) = 258 nm (192,800 L mol^−1^ cm^−1^), 315.5 nm (29,800 L mol^−1^ cm^−1^), 329 nm (29,600 L mol^−1^ cm^−1^), 367 nm (18,100 L mol^−1^ cm^−1^), 387 nm (14,200 L mol^−1^ cm^−1^). HRMS (ESI) calcd. for [C_45_H_32_N_2_O + H]^+^: 617.2586; Found: 617.2577.

#### 4-(1-benzyl-3-(4-cyanophenyl)-5-phenyl-1H-pyrrol-2-yl)phenyl 5-(dimethylamino)naphthalene-1-sulfonate (9)

According to the GP (variation A) compound **9** (42 mg, 61%) was obtained as a yellow solid, Mp 124°C.

^1^H NMR (300 MHz, CDCl_3_): δ 2.90 (s, 6 H), 4.97 (s, 2 H), 6.52 (m, 3 H), 6.80 (d, ^3^*J* = 8.37 Hz, 2 H), 6.93 (d, ^3^*J* = 8.79 Hz, 2 H), 7.08 (m, 5 H), 7.23 (m, 1 H), 7.33 (m, 7 H), 7.44 (dd, ^3^*J* = 7.4 Hz, ^3^*J* = 8.5 Hz, 1 H), 7.64 (dd, ^3^*J* = 7.4 Hz, ^3^*J* = 8.5 Hz, 1 H), 8.06 (dd, ^4^*J* = 1.3 Hz, ^3^*J* = 7.3 Hz, 1 H), 8.44 (d, ^3^*J* = 8.7 Hz, 1 H), 8.62 (d, ^3^*J* = 8.5 Hz, 1 H). ^13^C NMR (75 MHz, CDCl_3_): δ 45.7 (CH_3_), 48.8 (CH_2_), 108.5 (C_quat_), 109.3 (CH), 116.0 (CH), 119.6 (C_quat_), 122.2 (C_quat_), 122.7 (CH), 123.0 (CH), 126.1 (CH), 127.3 (CH), 127.8 (CH), 128.0 (CH), 128.6 (CH), 128.7 (CH), 128.8 (CH), 129.3 (CH), 129.3 (CH), 129.8 (C_quat_), 130.3 (C_quat_), 131.0 (C_quat_), 131.4 (CH), 131.6 (C_quat_), 132.0 (C_quat_), 132.1 (CH), 132.3 (CH), 132.5 (CH), 132.8 (C_quat_), 136.9 (C_quat_), 138.4 (C_quat_), 140.9 (C_quat_), 149.7 (C_quat_). MALDI-TOF [*m/z* (%)]: 660.17 ([M-H]^+^, 100). IR (KBr): $\tilde{\nu }$ [cm^−1^] = 2,924 (w), 2,222 (w), 1,602 (m), 1,570 (w), 1,508 (w), 1,452 (w), 1,369 (s), 1,307 (w), 1,193 (m), 1,174 (s), 1,145 (s), 1,049 (w), 1,018 (w), 943 (w), 860 (s), 844 (s). HRMS (ESI) calcd. for [C_42_H_33_N_3_O_3_S + H]^+^: 660.23154; Found: 660.23087.

#### 4-(1-benzyl-2-(4-(3-((9-(diethylamino)-5-oxo-5H-benzo[a]phenoxazin-2-yl)oxy)propoxy)phenyl)-5-phenyl-1h-pyrrol-3-yl)benzonitrile (10)

According to the GP (variation A) compound **10** (46mg, 59%) was obtained as a red solid, Mp 143°C.

^1^H NMR (600 MHz, acetone-d_6_): δ 1.26 (t, ^3^*J* = 7.1 Hz, 6 H), 2.36 (p, ^3^*J* = 6.2 Hz, 2 H), 3.58 (q, ^3^*J* = 7.1 Hz, 4 H), 4.29 (t, ^3^*J* = 6.2 Hz, 2 H), 4.42 (t, ^3^*J* = 6.2 Hz, 2 H), 5.16 (s, 2 H), 6.12 (s, 1 H), 6.60 (d, ^4^*J* = 2.7 Hz, 1 H), 6.68 (m, 3 H), 6.82 (dd, ^3^*J* = 9.1 Hz, ^4^*J* = 2.8 Hz, 2 H), 6.99 (d, ^3^*J* = 8.8 Hz, 2 H), 7.11 (m, 3 H), 7.17 (d, ^3^*J* = 8.8 Hz, 2 H), 7.27 (dd, ^3^*J* = 8.7 Hz, ^4^*J* = 2.6 Hz, 1 H), 7.31 (m, 1 H), 7.37 (m, 2 H), 7.40 (d, ^3^*J* = 8.7 Hz, 2 H), 7.46 (m, 2 H), 7.53 (d, ^3^*J* = 8.6 Hz, 1 H), 7.58 (d, ^3^*J* = 9.0 Hz, 1 H), 8.08 (d, ^4^*J* = 2.6 Hz, 1 H), 8.11 (d, ^3^*J* = 8.7 Hz, 1 H). ^13^C NMR (150 MHz, acetone-d_6_): δ 12.9 (CH_3_), 45.6 (CH_2_), 49.0 (CH_2_), 65.2 (CH_2_), 65.7 (CH_2_), 97.1 (CH), 105.4 (CH), 107.6 (CH), 108.8 (C_quat_), 109.9 (CH), 110.7 (CH), 111.5 (C_quat_), 115.7 (CH), 118.7 (CH), 119.7 (C_quat_), 122.1 (C_quat_), 125.1 (C_quat_), 125.5 (C_quat_), 126.6 (C_quat_), 126.6 (CH), 127.7 (CH), 128.2 (CH), 128.3 (CH), 128.4 (CH), 129.1 (CH), 129.4 (CH), 129.7 (CH), 131.9 (CH), 132.8 (CH), 133.3 (CH), 134.1 (C_quat_), 134.6 (C_quat_), 135.0 (C_quat_), 136.8 (C_quat_), 139.9 (C_quat_), 140.4 (C_quat_), 142.4 (C_quat_), 147.7 (C_quat_), 152.0 (C_quat_), 152.9 (C_quat_), 160.0 (C_quat_), 162.5 (C_quat_), 182.5 (C_quat_). MALDI-TOF [*m/z* (%)]: 801.3 ([M-H]^+^, 100). IR (KBr): $\tilde{\nu }$ [cm^−1^] = 2,965 (w), 2,926 (w), 2,222 (w), 1,732 (w), 1,709 (w), 1,620 (m), 1,595 (s), 1,580 (s), 1,516 (m), 1,495 (m), 1,466 (m), 1,406 (m), 1,339 (m), 1,314 (m), 1,254 (s), 1,223 (m), 1,177 (m), 1,111 (s), 1,080 (m), 1,026 (m), 966 (w), 907 (w), 876 (w), 827 (s), 795 (s). HRMS (ESI) calcd. for [C_53_H_44_N_4_O_4_ + H]^+^: 801.34353; Found: 801.34390.

#### *(E)*-2-(4-(4-((2-(4-(1-benzyl-3-(4-cyanophenyl)-5-phenyl-1h-pyrrol-2-yl)phenoxy)ethyl)-(methyl)amino)styryl)-3-cyano-5,5-dimethylfuran-2(5H)-ylidene)malononitrile (11)

According to the GP (variation B) compound **11** (78 mg, 39%) was obtained as a blue solid, Mp 135°C.

^1^H NMR (300 MHz, CDCl_3_): δ 1.76 (s, 6 H), 3.22 (s, 3 H), 3.91 (t, ^3^*J* = 5.2 Hz, 2 H), 4.19 (t, ^3^*J* = 5.2 Hz, 2 H), 5.05 (s, 2 H), 6.57 (s, 1 H), 6.66 (dd, ^3^*J* = 6.7 Hz, ^4^*J* = 2.8 Hz, 2 H), 6.79 (m, 5 H), 7.08 (d, ^3^*J* = 8.7 Hz, 2 H), 7.13 (m, 3 H), 7.24–7.39 (m, 7 H), 7.41 (d, ^3^*J* = 8.6 Hz, 2 H), 7.56 (d, ^3^*J* = 9.0 Hz, 2 H), 7.61 (d, ^3^*J* = 16.1 Hz, 1 H). ^13^C NMR (75 MHz, CDCl_3_): δ 26.9 (CH_3_), 39.8 (CH_3_), 48.5 (CH_2_), 51.9 (CH_2_), 65.4 (CH_2_), 97.1 (C_quat_), 108.0 (C_quat_), 109.1 (CH), 109.5 (CH), 111.5 (C_quat_), 112.7 (CH), 114.8 (CH), 119.6 (C_quat_), 119.65 (C_quat_), 119.7 (C_quat_), 119.8 (C_quat_), 121.6 (C_quat_), 122.7 (C_quat_), 125.4 (C_quat_), 126.0 (CH), 127.1 (CH), 127.6 (CH), 127.7 (CH), 128.5 (CH), 128.7 (CH), 129.2 (CH), 132.1 (CH), 132.3 (CH), 132.6 (CH), 133.0 (C_quat_), 133.1 (C_quat_), 136.2 (C_quat_), 138.9 (C_quat_), 141.4 (C_quat_), 148.2 (CH), 152.9 (C_quat_), 158.4 (C_quat_), 174.4 (C_quat_), 176.3 (C_quat._). MALDI-TOF [*m/z* (%)]: 769.3 ([M-H]^+^, 100). IR (KBr): $\tilde{\nu }$ [cm^−1^] = 2,960 (w), 2,222 (m), 1,599 (w), 1,558 (m), 1,516 (s), 1,464 (m), 1,435 (w), 1,371 (s), 1,276 (s), 1,244 (m), 1,190 (m), 1,170 (s), 1,150 (m), 1,105 (m), 1,072 (m), 1,031 (m), 1,016 (m), 966 (w), 937 (w), 839 (m), 815 (m), 795 (m). HRMS (ESI) calcd. for [C_51_H_40_N_6_O_2_ + Na]^+^: 791.31050; Found: 791.31045.

#### *(E)*-4-(1-benzyl-2-(4-(2-(methyl(4-(2-(4-methyl-3-oxo-3,4-dihydrochinoxalin-2-yl)vinyl)phenyl)amino)ethoxy)phenyl)-5-phenyl-1H-pyrrol-3-yl)benzonitrile (12)

According to the GP (variation B) compound **12** (70mg, 38%) was obtained as an orange solid, Mp 152°C.

^1^H NMR (300 MHz, CDCl_3_): δ 3.12 (s, 3 H), 3.73 (s, 3 H), 3.82 (t, ^3^*J* = 5.6 Hz, 2 H), 4.15 (m, 2 H), 5.06 (s, 2 H), 6.58 (s, 1 H), 6.66 (m, 2 H), 6.75 (d, ^3^*J* = 8.9 Hz, 2 H), 6.8 (d, ^3^*J* = 8.8 Hz, 2 H), 7.07 (d, ^3^*J* = 8.7 Hz, 2 H), 7.26–7.50 (m, 16 H), 7.62 (m, 2 H), 7.85 (dd, ^3^*J* = 8.0 Hz, ^4^*J* = 1.1 Hz, 1 H), 8.11 (d, ^3^*J* = 16.0 Hz, 1 H). ^13^C NMR (75 MHz, CDCl_3_): δ 29.3 CH_3_, 39.5 (CH_3_), 48.5 (CH_2_), 51.8 (CH_2_), 65.4 (CH_2_), 108.0 (C_quat_), 109.0 (CH), 112.0 (CH), 113.7 (CH), 114.8 (CH), 117.5 (CH), 119.7 (C_quat_), 121.4 (C_quat_), 123.9 (CH), 125.0 (C_quat_), 125.1 (C_quat_), 126.0 (CH), 127.1 (CH), 127.5 (CH), 127.6 (CH), 128.4 (CH), 128.6 (CH), 129.0 (CH), 129.2 (CH), 129.4 (CH), 129.9 (CH), 132.1 (CH), 132.5 (CH), 132.8 (C_quat_), 133.1 (C_quat_), 133.4 (C_quat_), 133.7 (C_quat_), 136.1 (C_quat_), 138.8 (C_quat_), 131.3 (C_quat_), 149.8 (C_quat_), 153.0 (C_quat_), 155.4 (C_quat_), 158.7 (C_quat_). MALDI-TOF [*m/z* (%)]:744.3 ([M-H]^+^, 100). IR (KBr): $\tilde{\nu }$ [cm^−1^] = 2,963 (w), 2,928 (w), 2,907 (w), 2,220 (w), 1,651 (w), 1,599 (m), 1,514 (w), 1,489 (w), 1,470 (w), 1,450 (w), 1,412 (w), 1,377 (w), 1,350 (w), 1,317 (w), 1,304 (w), 1,258 (s), 1,179 (m), 1,084 (s), 1,013 (s), 864 (m), 790 (s). HRMS (ESI) calcd. for [C_50_H_41_N_5_O_2_ + H]^+^: 744.33330; Found: 744.33373.

## Author contributions

The project was conceptualized by TM for the Ph.D. thesis of OG, who developed the synthetic approach and conducted the photophysical studies and their evaluation. BM performed all DFT and TD-DFT calculations and assigned the absorption transitions. Based upon the doctoral thesis of OG manuscript was written and corrected by TM and BM.

### Conflict of interest statement

The authors declare that the research was conducted in the absence of any commercial or financial relationships that could be construed as a potential conflict of interest. The handling editor declared a past co-authorship with one of the authors TM.

## Supplementary material

Data Sheet 1 This Supplementary Information file contains the ^1^H and ^13^C NMR spectra of compounds **1**, **2**, **8**–**12**, the absorption and emission spectra of compounds **2**, **8**–**12**, and the data and evaluation of the DFT and TD-DFT calculations on the structures **2**, **8**–**12**.
