# Supplementary material for: Diversity-Oriented Synthesis and Optical Properties of Bichromophoric Pyrrole-Fluorophore Conjugates
Source: Front Chem. 2018 Nov 27;6:579. doi: 10.3389/fchem.2018.00579 (PMC6277781; doi:10.3389/fchem.2018.00579)
Supplement: Data Sheet 1 — This Supplementary Information file contains the 1H and 13C NMR spectra of compounds 1, 2, 8–12, the absorption and emission spectra of compounds 2, 8–12, and the data and evaluation of the DFT and TD-DFT calculations on the structures 2, 8–12. [file Data_Sheet_1.pdf]

Supplementary Material

Diversity-oriented Synthesis and Optical Properties of Bichromophoric Pyrrole-Fluorophore Conjugates

Oliver Grotkopp,[1] Bernhard Mayer,[1] and Thomas J. J. Müller[1]*

[1]Institut für Organische Chemie und Makromolekulare Chemie, Heinrich-Heine-Universität Düsseldorf, Universitätsstrasse 1, D-40225 Düsseldorf, Germany

*** Correspondence:** Thomas J. J. Müller: ThomasJJ.Mueller@hhu.de

Table of contents

1. NMR Spectra 2
1.1. Pyrrole **1** 2
1.2. Pyrrole **2** 3
1.3. Bichromophore **8** 4
1.4. Bichromophore **9** 5
1.5. Bichromophore **10** 6
1.6. Bichromophore **11** 7
1.7. Bichromophore **12** 8
2. UV/Vis and Fluorescence Spectra 9
2.1. Bichromophore **8** 9
2.2. Bichromophore **9** 10
2.3. Bichromophore **10** 12
2.4. Bichromophore **11** 13
2.5. Bichromophore **12** 14
3. Calculations on the Structures of Pyrrole **2** and Bichromophores **8-12** 15
3.1. Pyrrole **2** 15
3.2. Pyrrole-anthracene bichromophore **8** 20
3.3. Pyrrole-dansyl bichromophore **9** 24
3.4. Pyrrole-Nile red bichromophore **10** 28
3.5. Pyrrole-3-cyano-5,5-dimethylfuran-2(5*H*)-ylidene)malononitrile-styryl bichromophore **11** 32
3.6. Pyrrole-quinoxalinyl-styryl bichromophore **12** 36
3.7. Assignment of the TD-DFT calculated transitions 40

1. NMR Spectra

1.1. Pyrrole 1

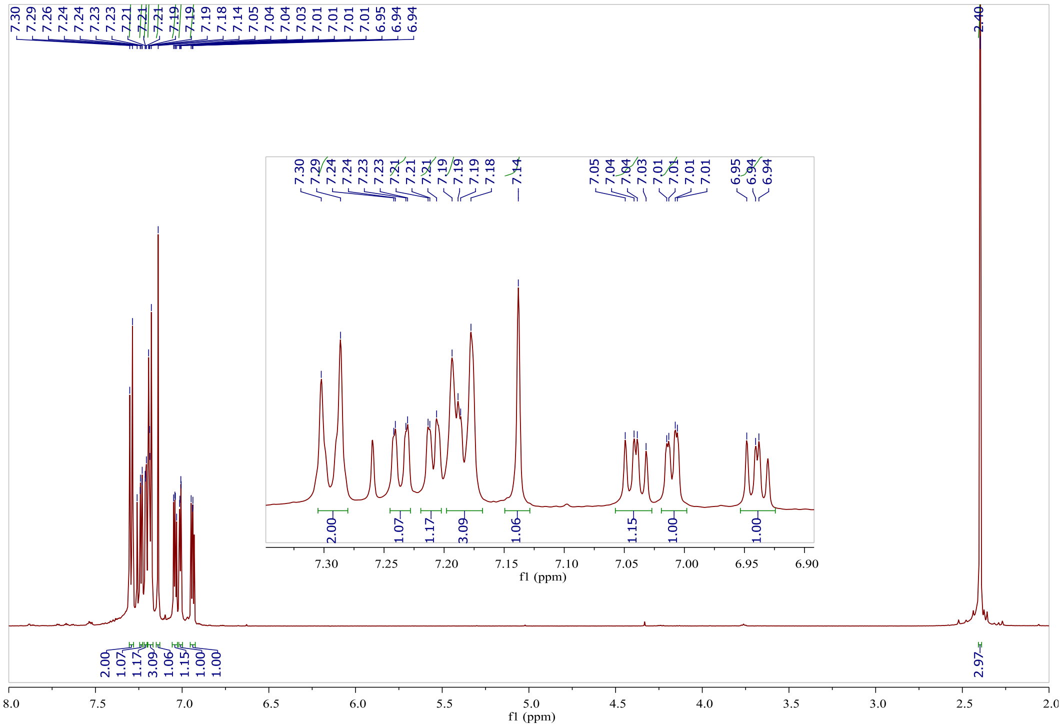

^{1}H NMR (500 MHz, $CDCl_3$) of compound **1** (recorded at T = 293 K).

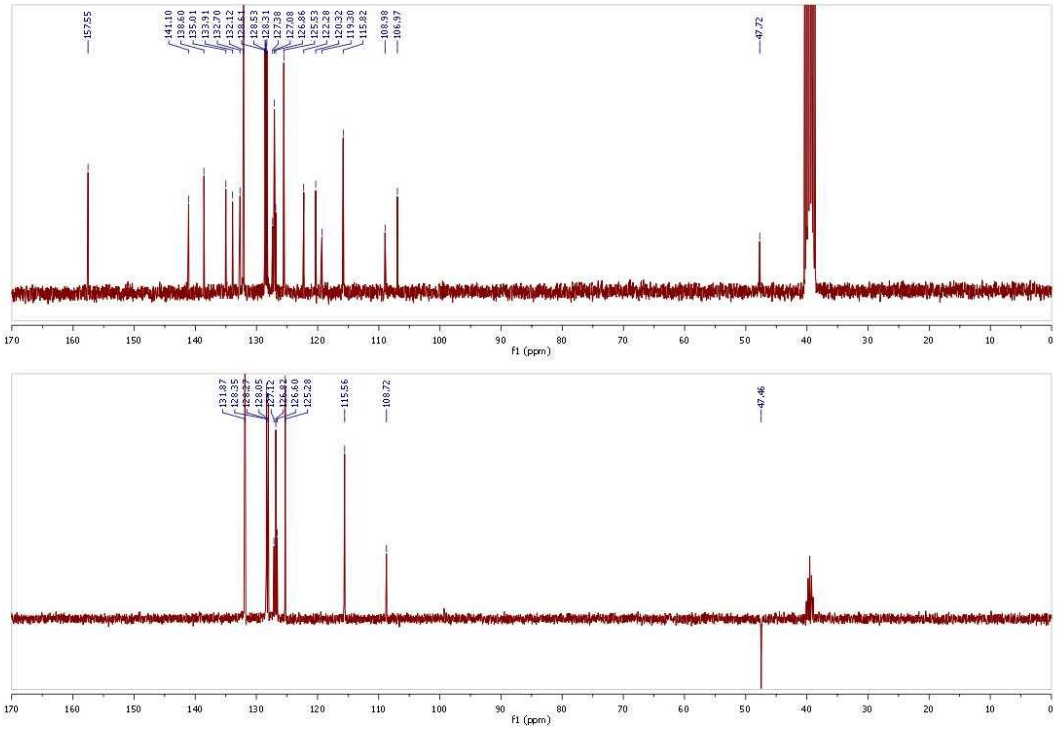

^{13}C NMR and 135 DEPT (126 MHz, $CDCl_3$) of compound **1** (recorded at T = 293 K).

1.2. Pyrrole 2

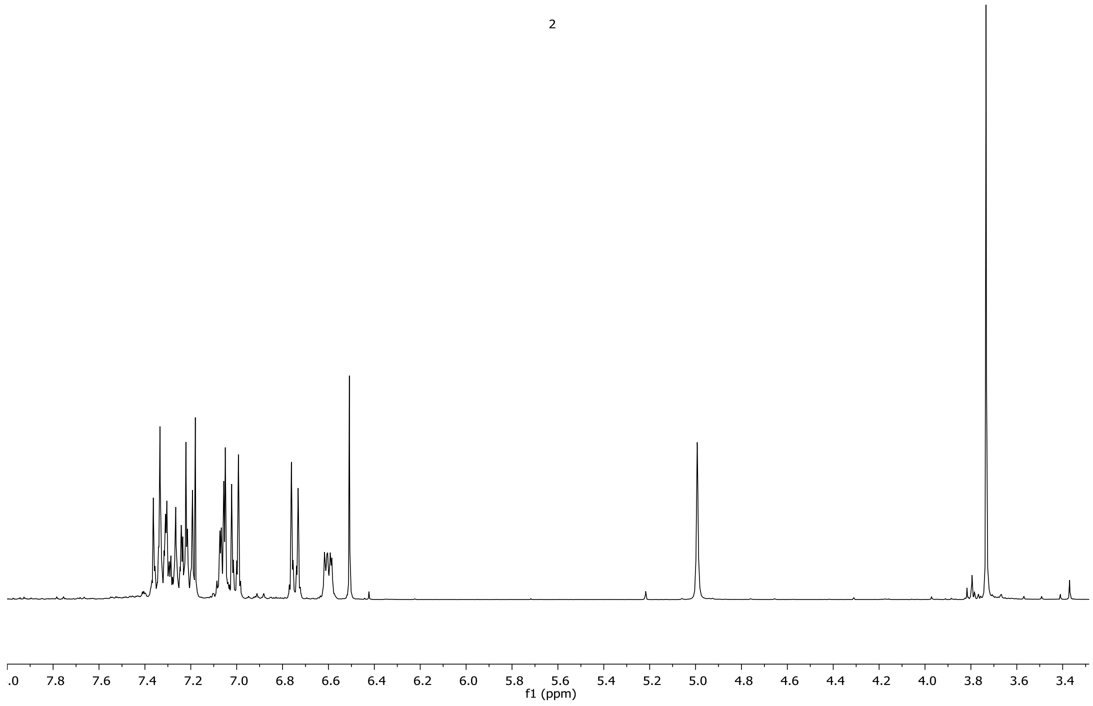

^{1}H NMR (300 MHz, $CDCl_3$) of compound **2** (recorded at T = 293 K).

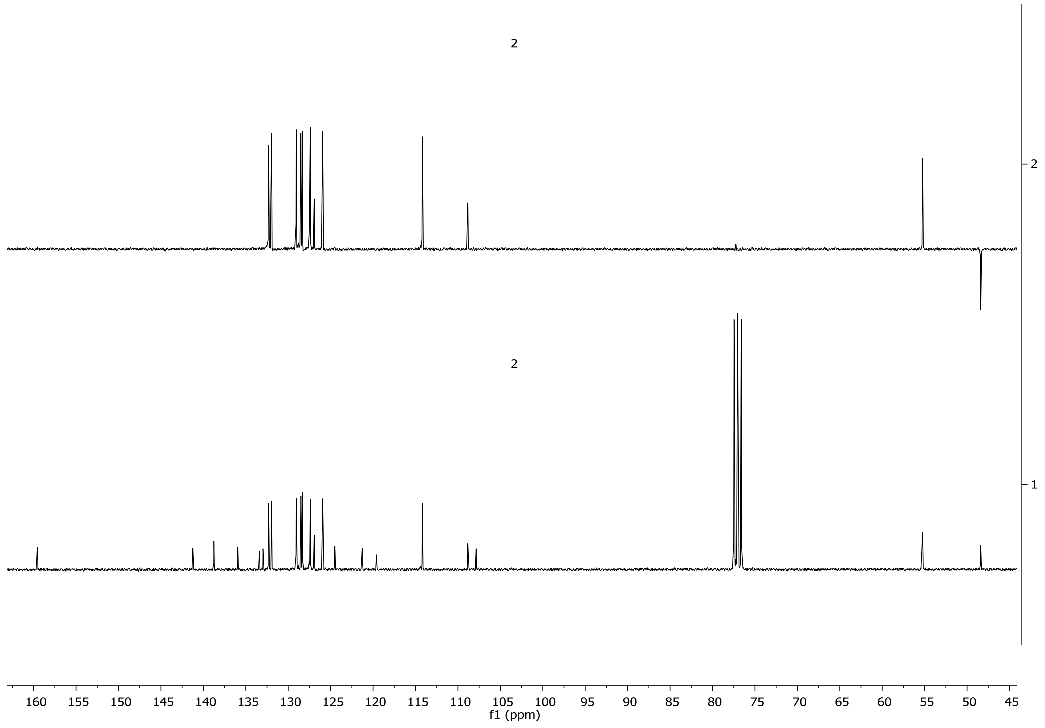

^{13}C NMR and 135 DEPT (75 MHz, $CDCl_3$) of compound **2** (recorded at T = 293 K).

1.3. Bichromophore 8

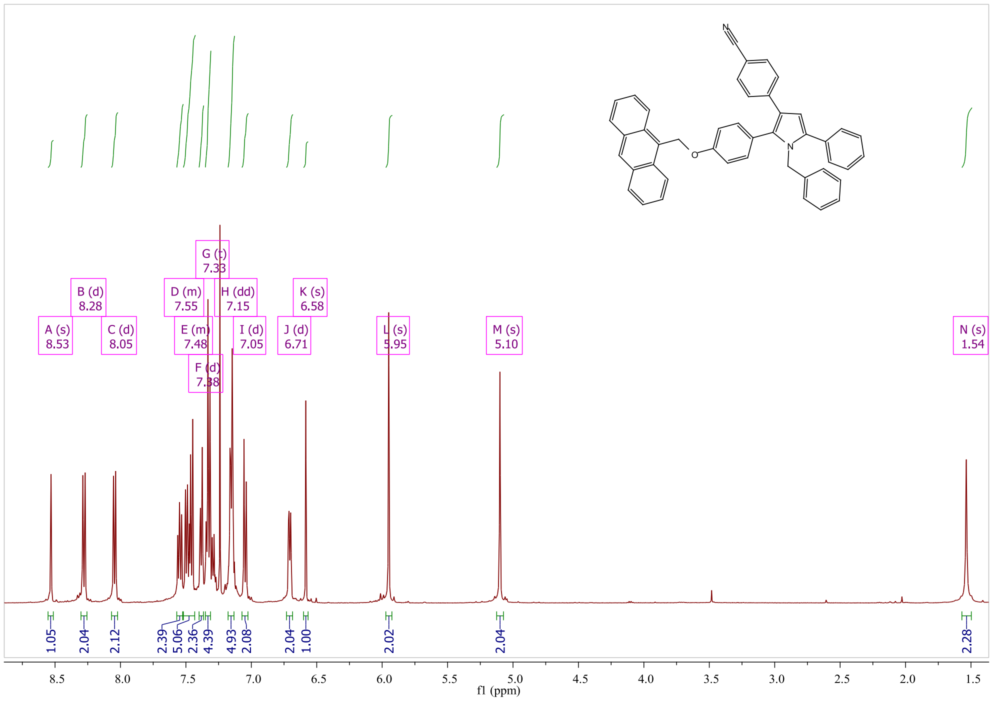

^{1}H NMR (500 MHz, $CDCl_3$) of compound **8** (recorded at T = 293 K).

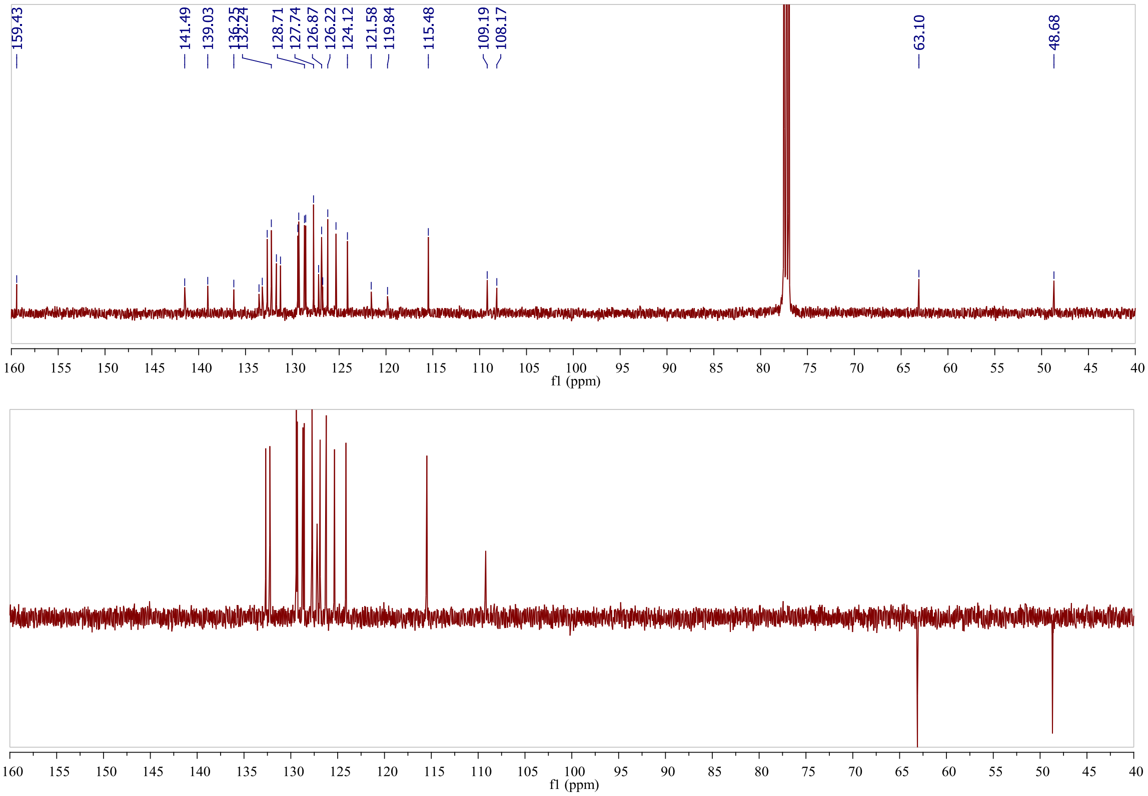

^{13}C NMR and 135 DEPT (126 MHz, $CDCl_3$) of compound **8** (recorded at T = 293 K).

1.4. Bichromophore 9

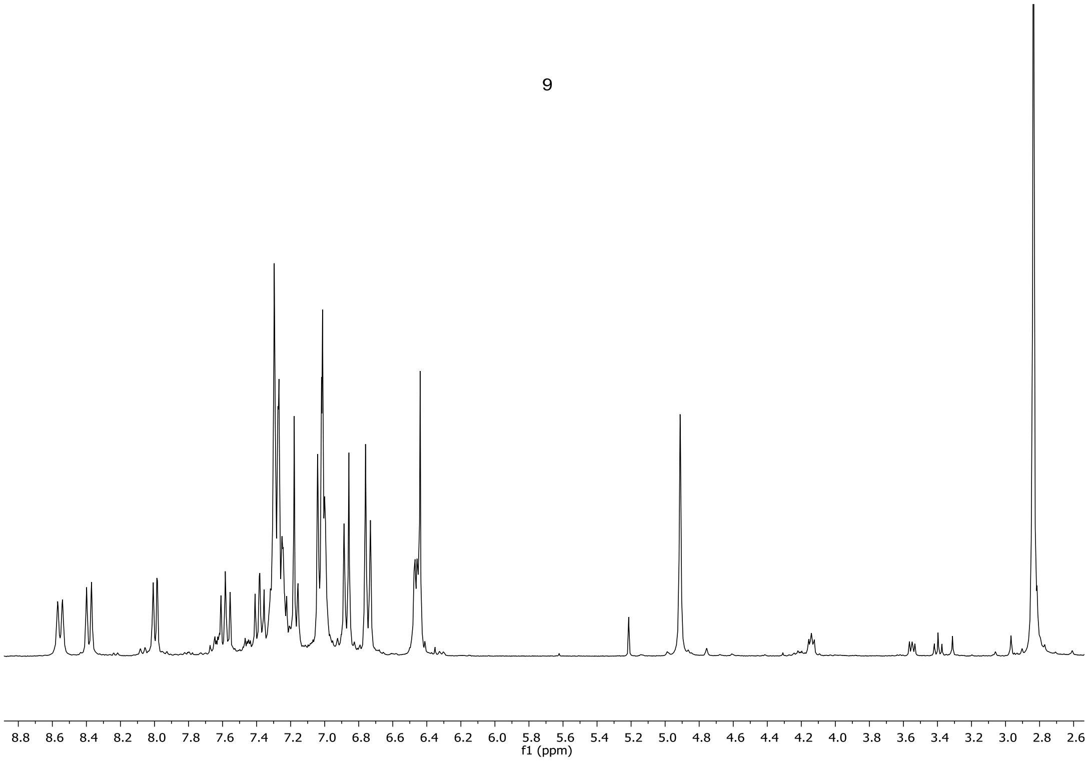

^{1}H NMR (300 MHz, $CDCl_3$) of compound **9** (recorded at T = 293 K).

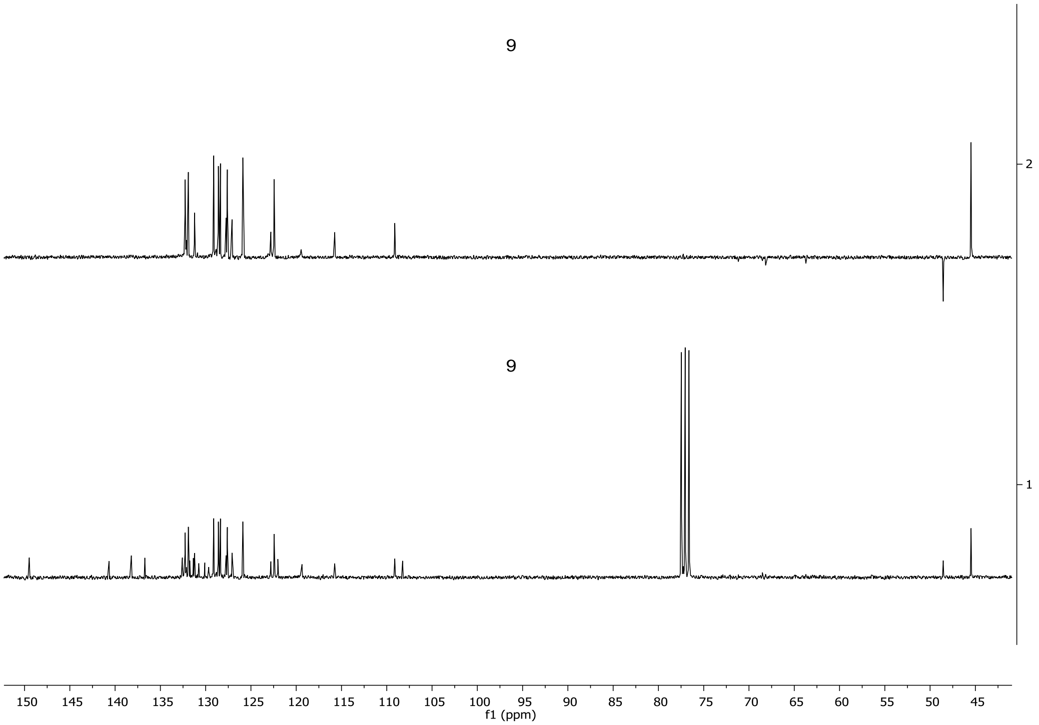

^{13}C NMR and 135 DEPT (75 MHz, $CDCl_3$) of compound **9** (recorded at T = 293 K).

1.5. Bichromophore 10

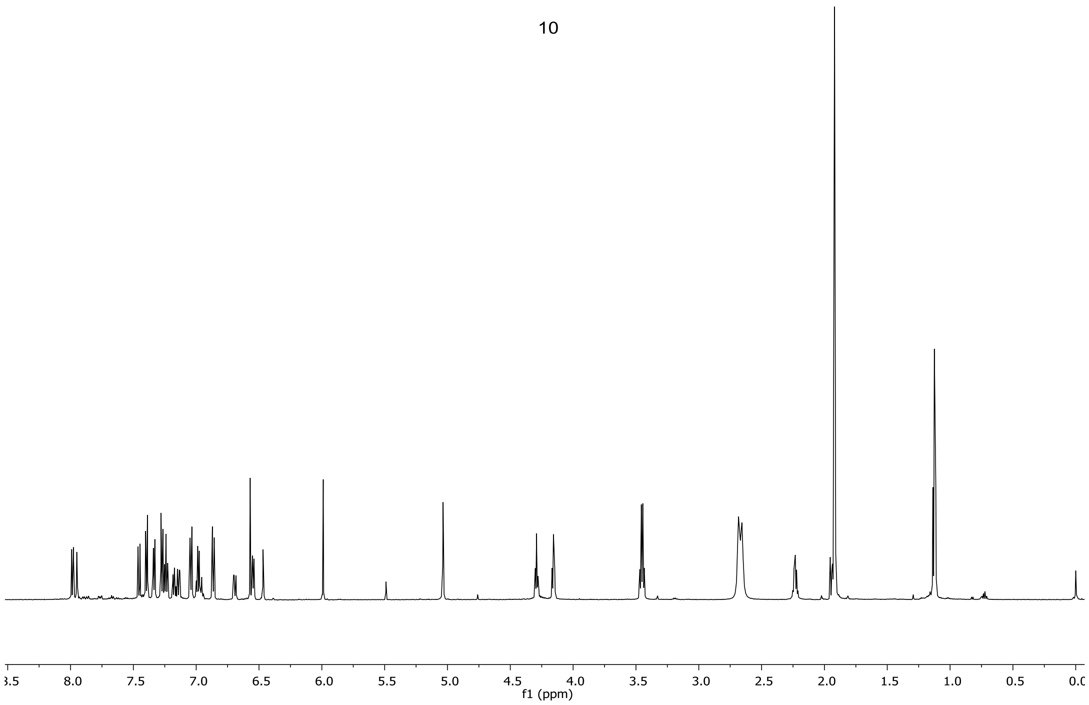

^{1}H NMR (600 MHz, $CDCl_3$) of compound **10** (recorded at *T* = 293 K).

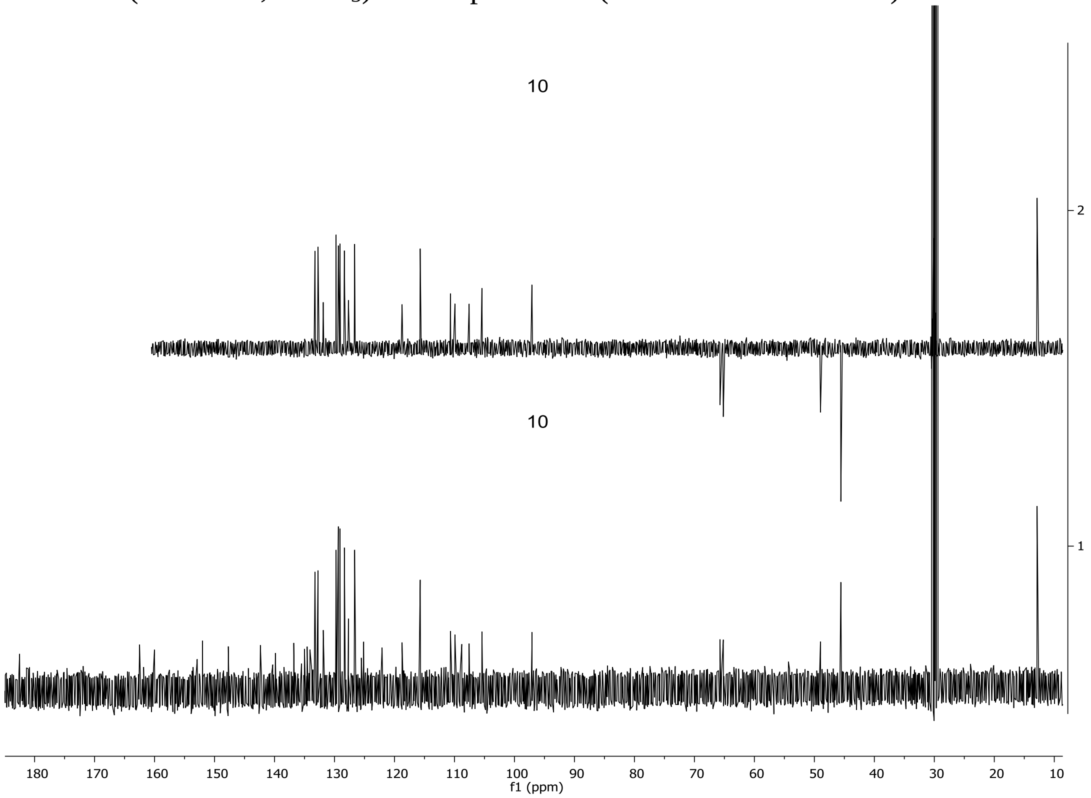

^{13}C NMR and 135 DEPT (135 MHz, $CDCl_3$) of compound **10** (recorded at *T* = 293 K).

1.6. Bichromophore 11

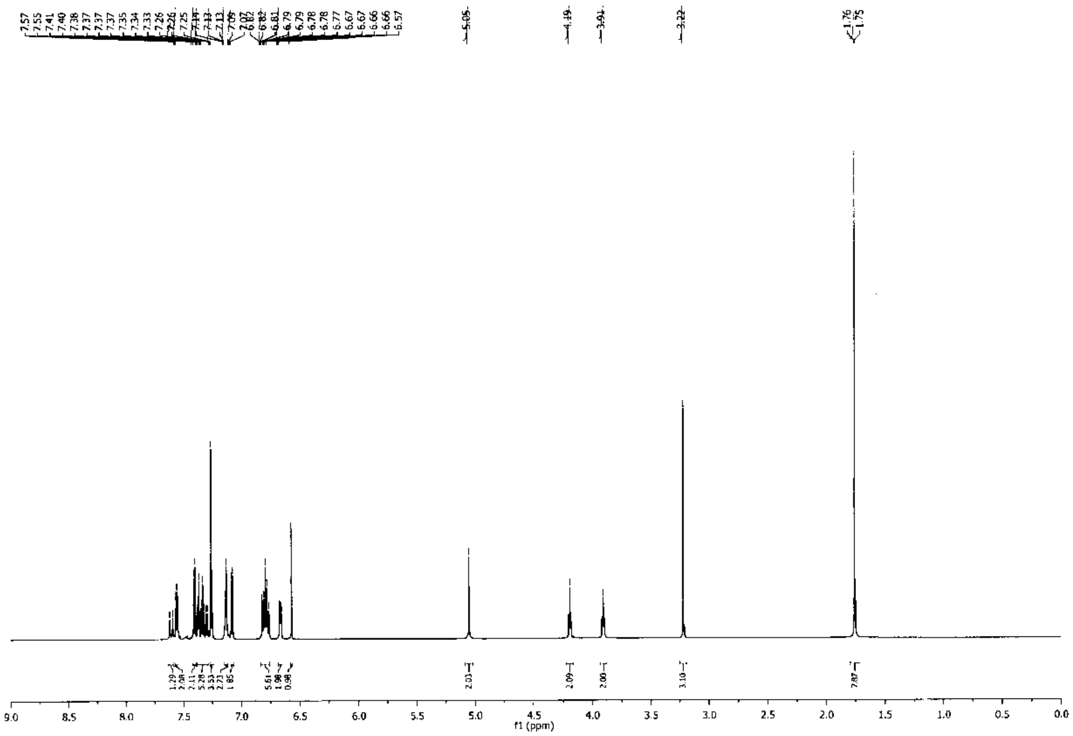

^{1}H NMR (300 MHz, $CDCl_3$) of compound **11** (recorded at T = 293 K).

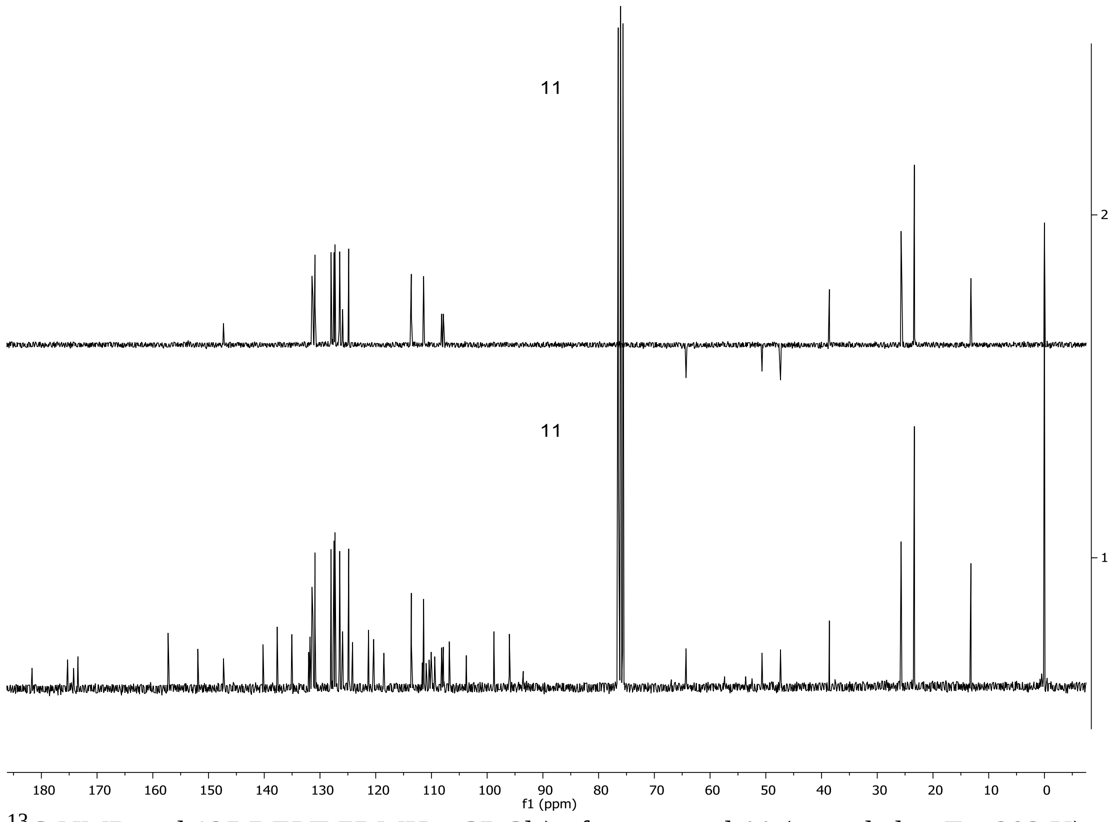

^{13}C NMR and 135 DEPT 75 MHz, $CDCl_3$) of compound **11** (recorded at T = 293 K).

1.7. Bichromophore 12

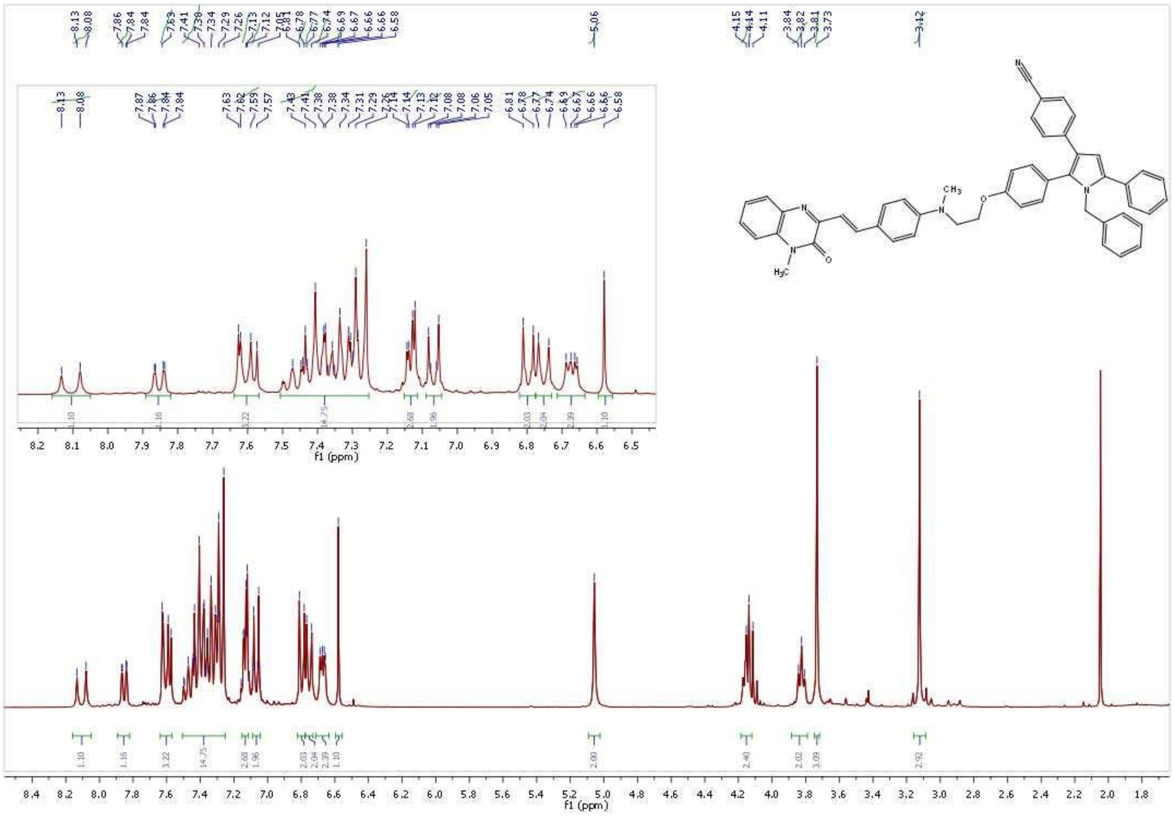

^{1}H NMR (300 MHz, $CDCl_3$) of compound **12** (recorded at T = 293 K).

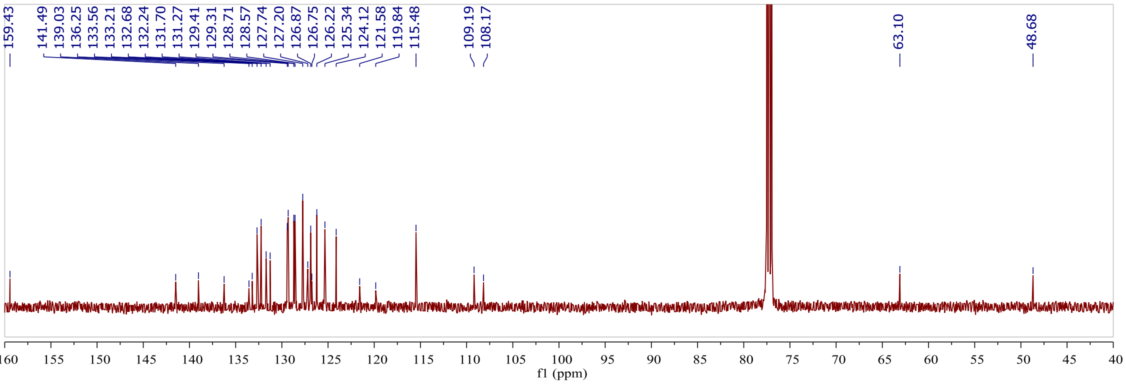

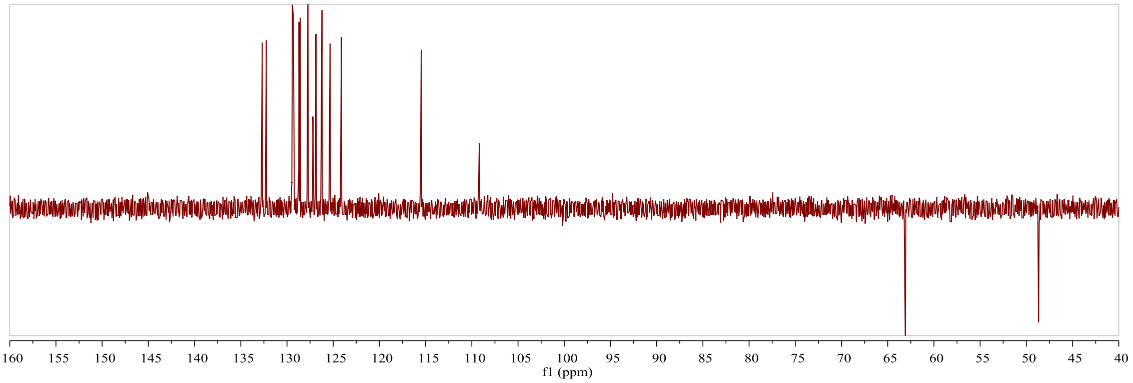

^{13}C NMR and 135 DEPT (75 MHz, $CDCl_3$) of compound **12** (recorded at T = 293 K).

2. UV/Vis and Fluorescence Spectra

2.1. Bichromophore 8

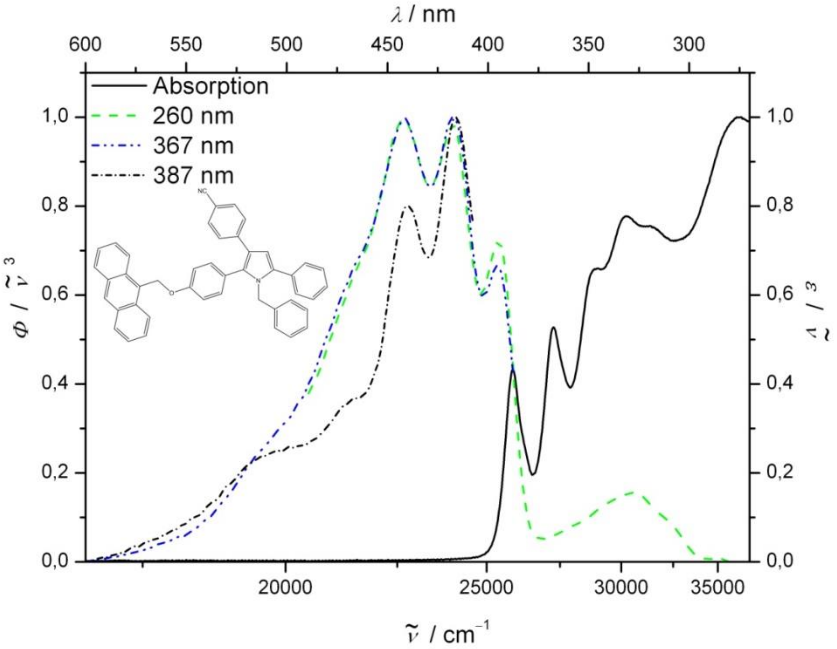

Figure S1. Normalized UV/Vis and emission spectra of bichromophore **8** (λ_{exc} = 260, 367 and 387 nm) (recorded in CH_2Cl_2, *T* = 298 K).

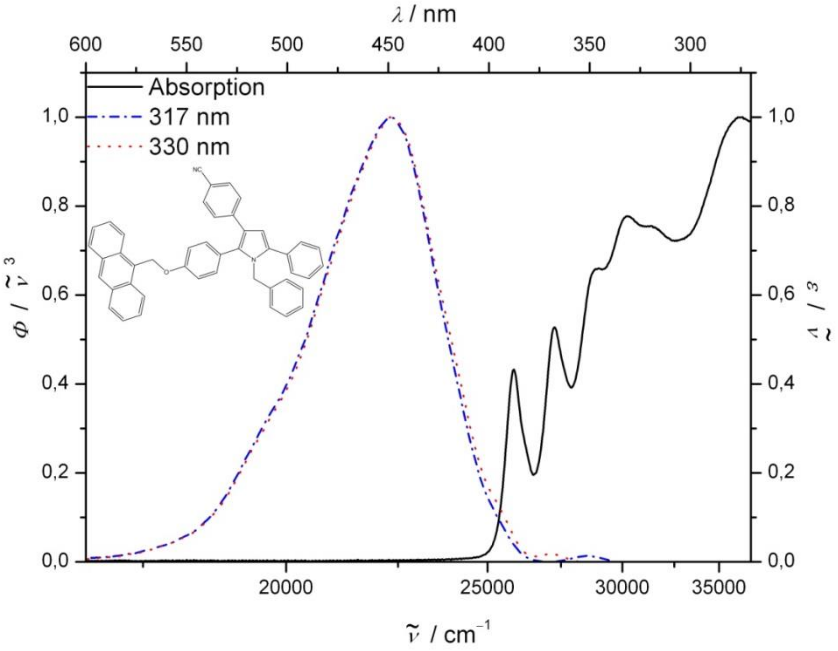

Figure S2. Normalized UV/Vis and emission spectra of bichromophore **8** (λ_{exc} = 317 and 330 nm) (recorded in CH_2Cl_2, *T* = 298 K).

2.2. Bichromophore 9

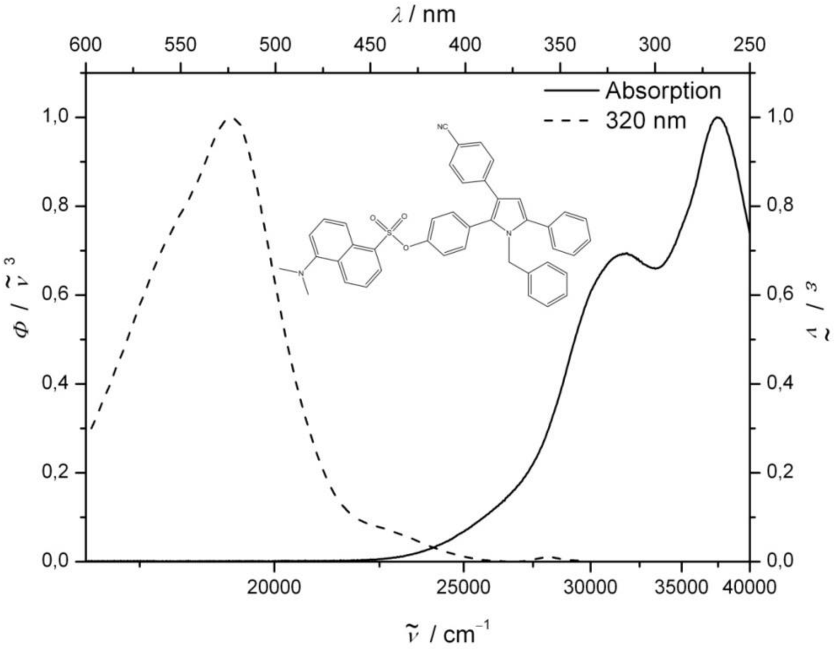

Figure S3. Normalized UV/Vis and emission spectra of bichromophore **9** (recorded in CH_2Cl_2, T = 298 K).

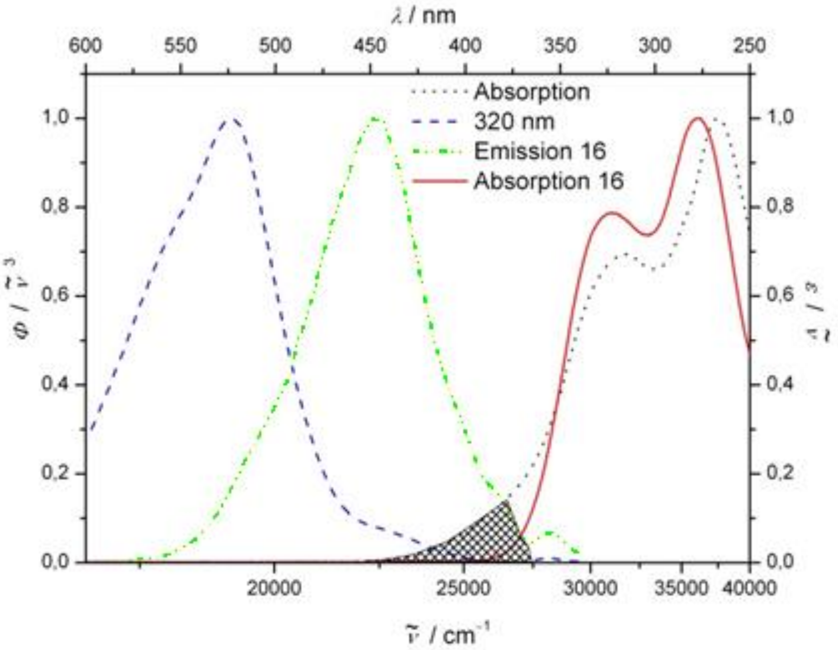

Figure S4. Normalized UV/Vis and emission spectra of bichromophore **9** and pyrrole **2** (recorded in CH_2Cl_2, T = 298 K).

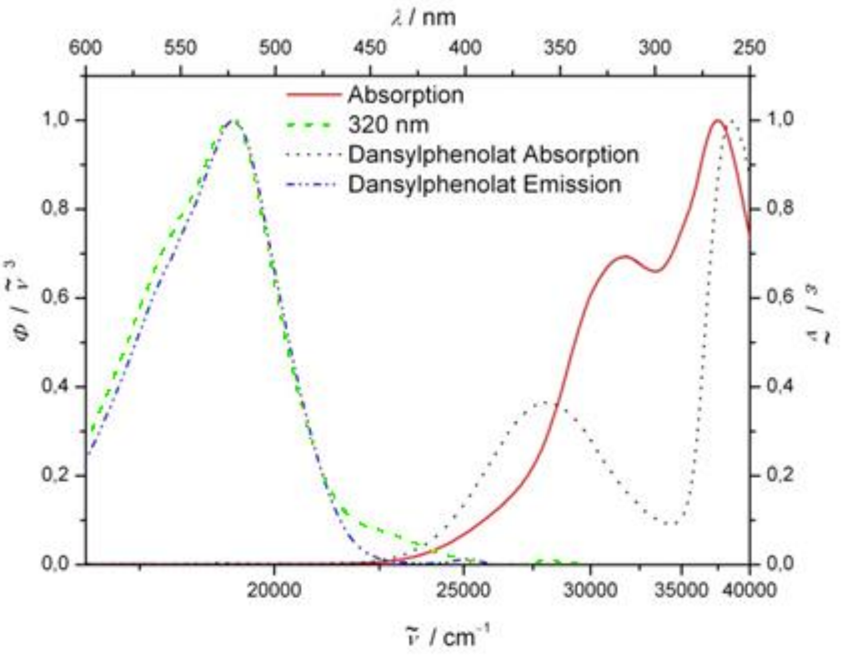

Figure S5. Normalized UV/Vis and emission spectra of bichromophore **9** and dansyl phenolate (recorded in CH_2Cl_2, T = 298 K).

2.3. Bichromophore 10

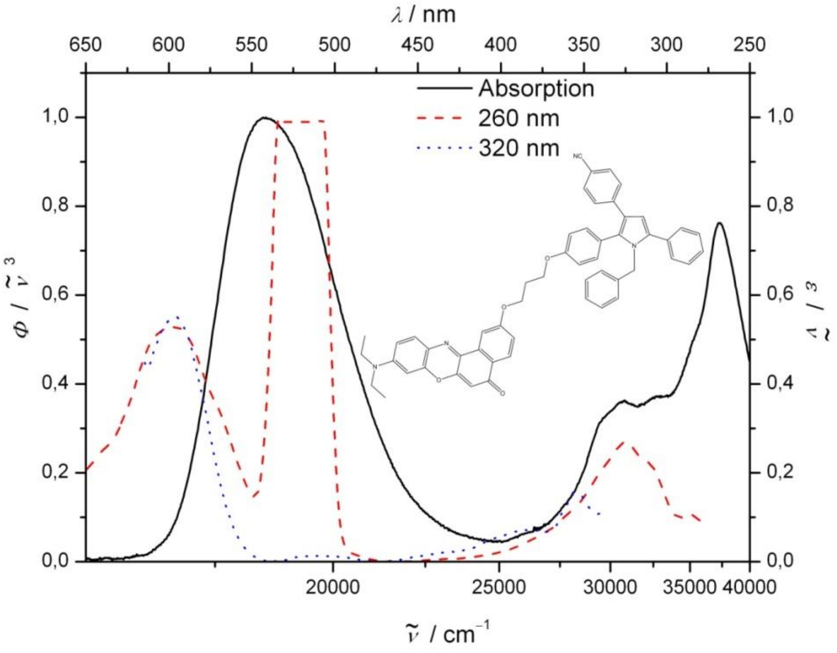

Figure S6. Normalized UV/Vis and emission spectra of bichromophore **10** (λ_{exc} = 260 and 320 nm) (recorded in CH_2Cl_2, T = 298 K).

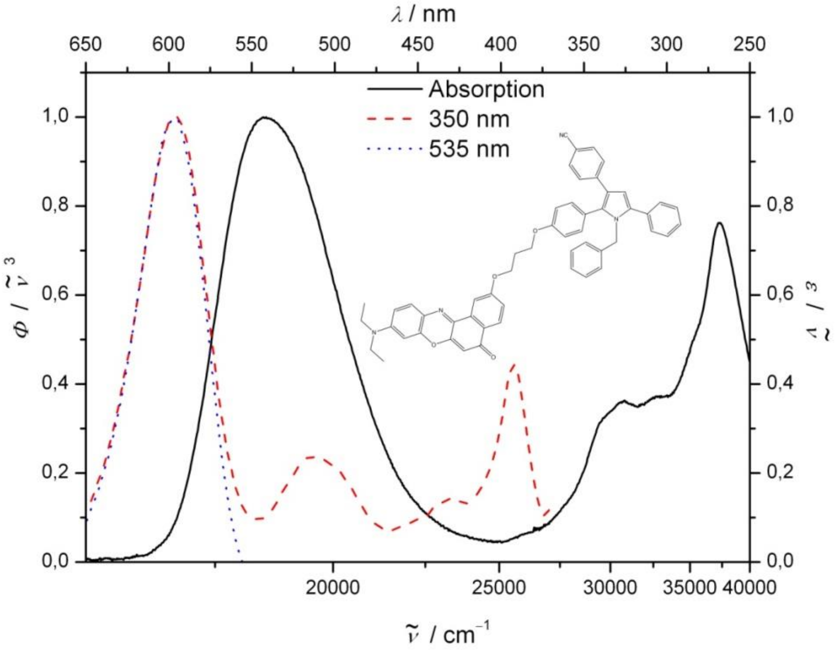

Figure S7. Normalized UV/Vis and emission spectra of bichromophore **10** (λ_{exc} = 350 and 535 nm) (recorded in CH_2Cl_2, T = 298 K).

2.4. Bichromophore 11

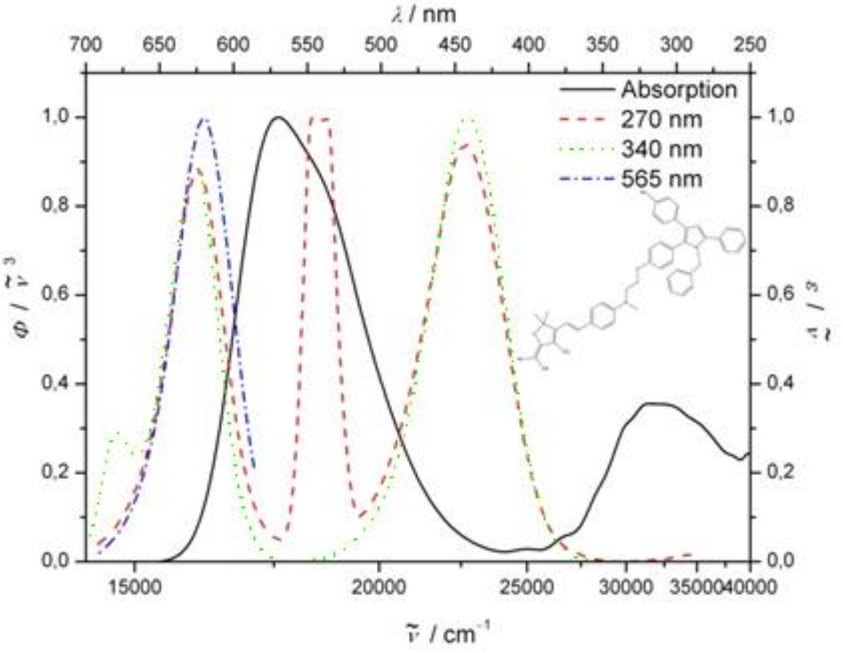

Figure S8. Normalized UV/Vis and emission spectra of bichromophore **11** (λ_{exc} = 270, 340, 565 nm) (recorded in CH_2Cl_2, T = 298 K).

2.5. Bichromophore 12

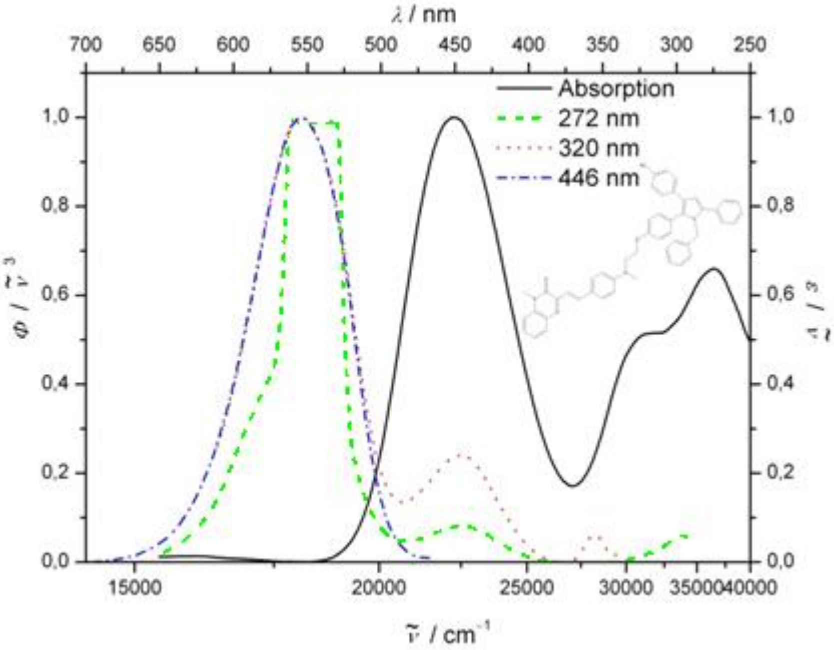

Figure S9. Normalized UV/Vis and emission spectra of bichromophore **12** (λ_{exc} = 272, 320, 446 nm) (recorded in CH_2Cl_2, T = 298 K).

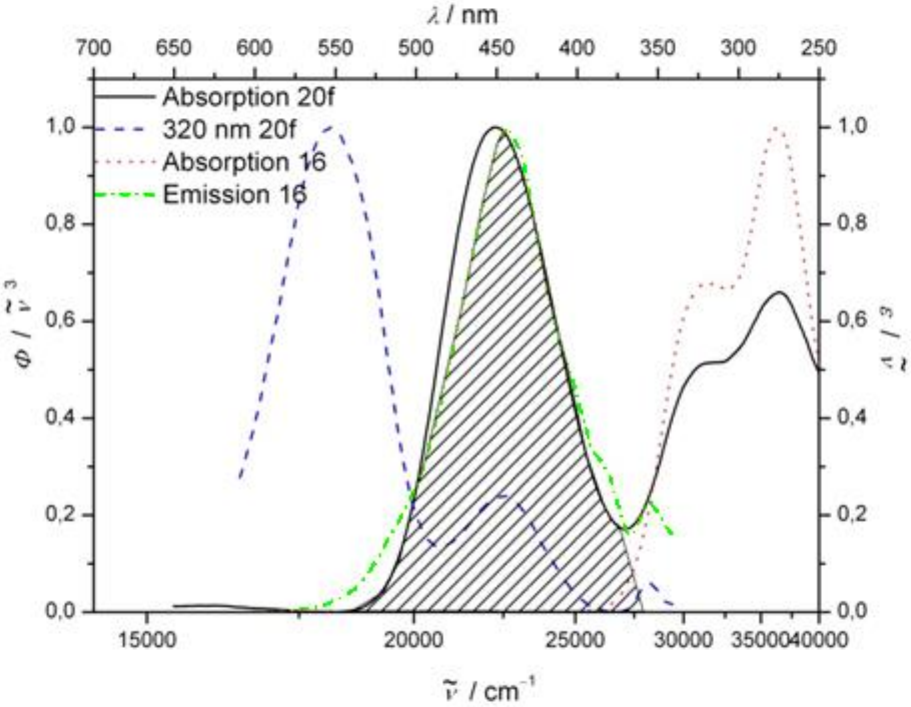

Figure S10. Normalized UV/Vis and emission spectra of bichromophore **12** and pyrrole **2** (recorded in CH_2Cl_2, T = 298 K). The shaded area corresponds to the prerequisite conditions for Förster resonance energy transfer.

3. Calculations on the Structures of Pyrrole 2 and Bichromophores 8-12

3.1. Pyrrole 2

B3LYP 6-311G SCRF (Solvent = Dichloromethane) optimized S_0 ground state xyz-coordinates**

N	-1.294838	-0.113343	-0.676456
C	0.074397	-0.149095	-0.465479
C	0.428659	-1.467703	-0.178994
C	-0.774670	-2.233529	-0.201827
C	-1.819608	-1.393673	-0.516952
H	-0.852924	-3.305098	-0.101171
C	-3.230839	-1.752048	-0.737954
C	-5.894458	-2.553663	-1.166082
C	-3.922587	-1.362338	-1.897217
C	-3.898638	-2.559308	0.196223
C	-5.214800	-2.959792	-0.018640
C	-5.242592	-1.753823	-2.104108
H	-3.415119	-0.774097	-2.653330
H	-3.380792	-2.862702	1.098740
H	-5.712817	-3.581925	0.716515
H	-5.757779	-1.444766	-3.006637
H	-6.921178	-2.860049	-1.329963
C	1.769631	-2.020452	0.048728
C	4.313168	-3.160175	0.486247
C	1.936163	-3.122249	0.909317
C	2.913865	-1.510199	-0.592630
C	4.165934	-2.064207	-0.378767
C	3.182169	-3.687676	1.128388
H	1.075103	-3.529547	1.425084
H	2.817289	-0.678795	-1.277762
H	5.032941	-1.659626	-0.886279
H	3.287682	-4.531470	1.799053
C	0.911282	1.066596	-0.519547
C	2.553436	3.357410	-0.627293
C	1.643532	1.486183	0.604103
C	1.025655	1.820906	-1.692393
C	1.831535	2.957401	-1.756221
C	2.453522	2.608597	0.553766
H	1.569164	0.924230	1.527479

H	0.493010	1.508837	-2.584060
H	1.894670	3.508942	-2.684192
H	3.015547	2.931928	1.421778
C	-2.088536	1.118031	-0.667505
H	-1.637208	1.832552	-1.355140
H	-3.072285	0.874536	-1.068429
C	-2.248734	1.760436	0.702201
C	-2.659690	3.003220	3.185151
C	-2.167157	1.023555	1.886032
C	-2.534065	3.127725	0.776546
C	-2.741496	3.745537	2.007570
C	-2.369893	1.641893	3.119622
H	-1.939664	-0.035339	1.848585
H	-2.590739	3.714465	-0.134914
H	-2.958673	4.807109	2.047262
H	-2.299916	1.057110	4.030070
H	-2.815659	3.482893	4.144712
O	3.370681	4.443446	-0.575406
C	3.514528	5.247085	-1.746857
H	4.201212	6.045853	-1.474247
H	2.556873	5.679563	-2.052556
H	3.938220	4.670711	-2.574974
C	5.600666	-3.735082	0.707459
N	6.644168	-4.200980	0.886501

Zero-point correction =	0.463982 (Hartree/particle)
Sum of electronic and zero-point Energies =	-1380.347017 a.u., -866167.7531675 kcal/mol
Sum of electronic and thermal Energies =	-1380.318660 a.u., 866149.9591499999 kcal/mol
Sum of electronic and thermal Enthalpies =	-1380.317716 a.u., 866149.36679 kcal/mol
Sum of electronic and thermal Free Energies =	-1380.410112 a.u., 866207.34528 kcal/mol

B3LYP 6-311G SCRF (Solvent = Dichloromethane) optimized S_1 state xyz-coordinates**

B3LYP 6-311G** TD (NStates = 10, Root = 1) SCRF (Solvent = Dichloromethane)

N	-1.421841	-0.007227	-0.703358
C	-0.036190	-0.102017	-0.464119
C	0.266103	-1.467547	-0.118804

C	-0.956273	-2.140830	-0.114419
C	-1.980972	-1.250834	-0.471948
H	-1.089350	-3.205186	-0.001450
C	-3.382761	-1.587890	-0.673568
C	-6.050899	-2.361073	-1.075860
C	-4.137016	-1.042440	-1.731451
C	-3.989416	-2.542119	0.166338
C	-5.311633	-2.917415	-0.030951
C	-5.456820	-1.428663	-1.927453
H	-3.673898	-0.349116	-2.422401
H	-3.422767	-2.968960	0.984903
H	-5.768091	-3.643281	0.631359
H	-6.020435	-1.010375	-2.752885
H	-7.081462	-2.657899	-1.230419
C	1.568839	-2.105259	0.044930
C	4.087205	-3.425176	0.375137
C	1.740827	-3.152459	0.996134
C	2.703933	-1.756068	-0.750313
C	3.913906	-2.388777	-0.596159
C	2.944834	-3.790525	1.168237
H	0.902754	-3.437496	1.624180
H	2.606564	-0.999229	-1.518955
H	4.751134	-2.110187	-1.226090
H	3.043313	-4.573603	1.910799
C	0.861285	1.004579	-0.595565
C	2.633354	3.203359	-0.774320
C	2.001754	1.110504	0.255791
C	0.675505	2.031014	-1.558627
C	1.540427	3.103592	-1.656307
C	2.848410	2.189052	0.181666
H	2.177796	0.345956	0.998952
H	-0.125323	1.948507	-2.280666
H	1.377944	3.847408	-2.423606
H	3.695142	2.282248	0.850098
C	-2.190199	1.246237	-0.522237
H	-1.947242	1.946747	-1.318174
H	-3.239162	0.985468	-0.648846
C	-1.998896	1.908997	0.831061

C	-1.772537	3.188743	3.316538
C	-2.007758	1.165228	2.015702
C	-1.873271	3.298206	0.906294
C	-1.765628	3.936593	2.141282
C	-1.891661	1.800712	3.249736
H	-2.102311	0.085760	1.979428
H	-1.857422	3.886091	-0.005469
H	-1.668908	5.015589	2.182813
H	-1.896066	1.211394	4.159638
H	-1.682540	3.681949	4.277584
O	3.525780	4.207077	-0.775143
C	3.386665	5.275793	-1.722170
H	4.212672	5.952410	-1.518600
H	2.437755	5.799275	-1.581623
H	3.461328	4.901002	-2.745935
C	5.323530	-4.063176	0.541310
N	6.355334	-4.597506	0.680675

Zero-point correction =	0.461236 (Hartree/particle)
Sum of electronic and zero-point Energies =	-1380.240534 a.u., -866100.935085 kcal/mol
Sum of electronic and thermal Energies =	-1380.211905 a.u., 866082.9703874999 kcal/mol
Sum of electronic and thermal Enthalpies =	-1380.210961 a.u., 866082.3780275 kcal/mol
Sum of electronic and thermal Free Energies =	-1380.303487 a.u., 866140.4380925 kcal/mol

PBEh1PBE 6-311G** TD = (NStates = 10, root = 1) SCRF = (Solvent = Dichloromethane)

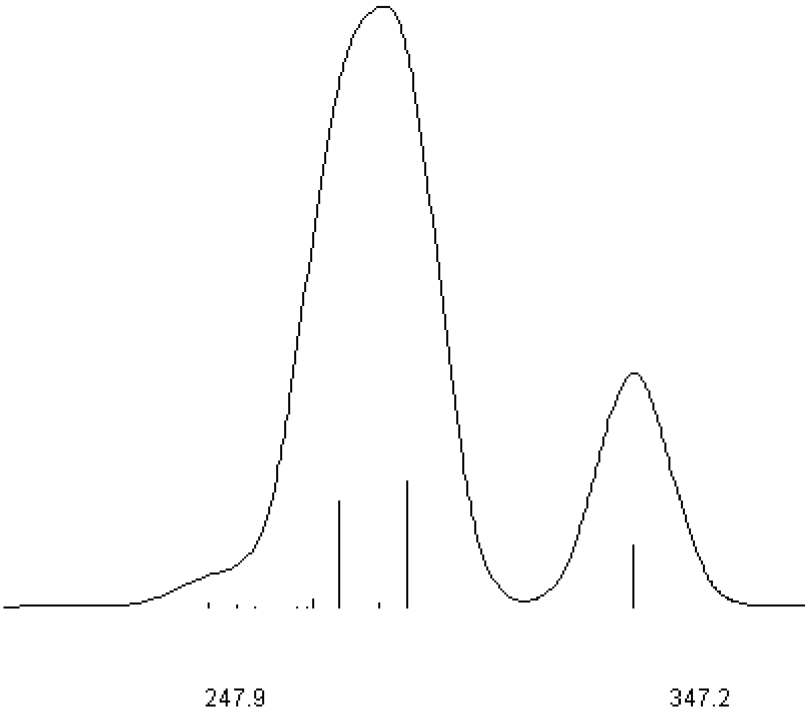

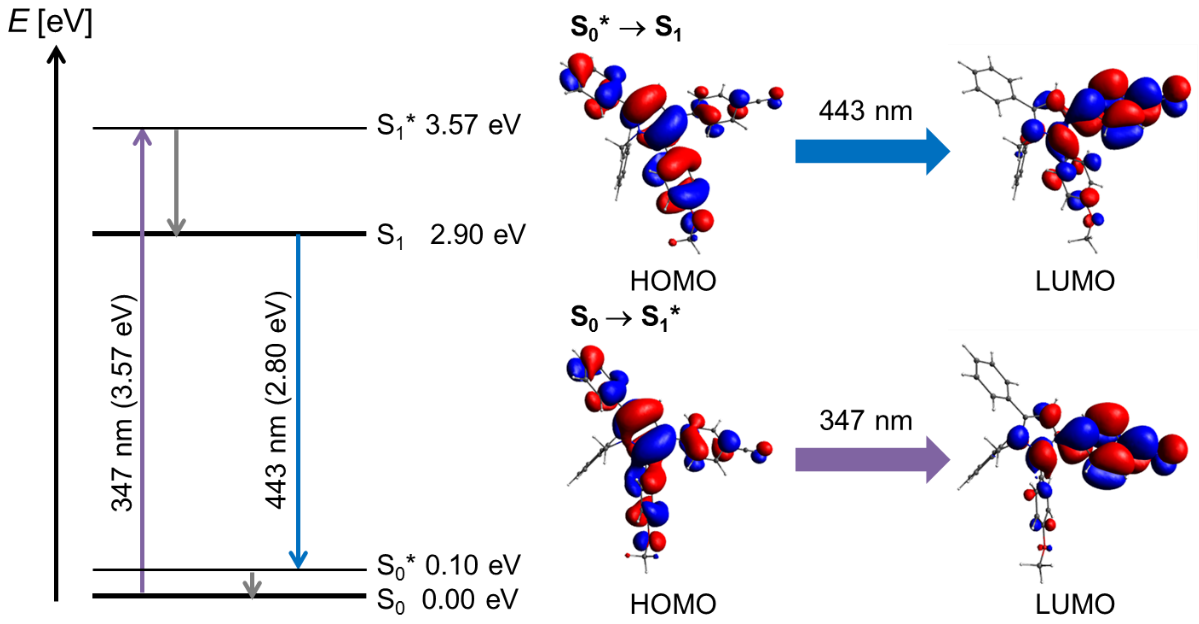

Figure S11. Jablonski diagram of compound **2** and assignment of the FMO-transitions in the longest wavelength absorption band and the emission band ($E(S_0)$ = 0 eV; PBEh1PBE 6-311G** IEFPCM CH_2Cl_2, isosurface value at 0.03 a.u.).

3.2. Pyrrole-anthracene bichromophore 8

B3LYP 6-311G SCRF (Solvent = Dichloromethane) optimized S_0 ground state xyz-coordinates**

H	-8.385956	3.742604	-1.290061
C	-7.744237	2.918976	-0.999382
H	-9.292550	1.454249	-1.098142
C	-8.246390	1.653677	-0.892034
C	-5.535292	2.138203	-0.362223
C	-7.410140	0.557671	-0.513093
C	-6.366520	3.157995	-0.736948
C	-6.015092	0.795241	-0.221603
C	-7.918759	-0.739104	-0.425488
H	-5.971390	4.162128	-0.841020
H	-4.489278	2.354665	-0.192244
C	-7.108329	-1.822737	-0.082679
H	-8.969030	-0.910764	-0.639266
C	-7.635201	-3.150360	-0.023464
C	-5.712921	-1.598678	0.212895
H	-3.856250	-2.626658	0.715649
C	-5.193538	-0.284507	0.174603
C	-6.836026	-4.211743	0.292858
H	-8.687758	-3.295961	-0.241663
H	-7.244499	-5.214888	0.332298
C	-5.455294	-4.002352	0.564393
H	-4.824316	-4.852179	0.798813
C	-4.914553	-2.746171	0.526600
C	-3.755744	-0.041179	0.555203
H	-3.615414	0.968987	0.943406
H	-3.437132	-0.734532	1.336734
O	-2.933575	-0.227306	-0.615999
C	-1.584918	-0.069664	-0.497838
C	1.222360	0.200836	-0.476196
C	-0.921500	0.282880	0.681532
C	-0.842111	-0.283498	-1.667485
C	0.536816	-0.146428	-1.653879
C	0.466834	0.410662	0.680997
H	-1.464897	0.451733	1.600991
H	-1.369494	-0.546700	-2.576483

H	1.091231	-0.292284	-2.574373
H	0.970256	0.673573	1.603764
C	2.689540	0.368909	-0.463972
N	3.553539	-0.674285	-0.755589
C	4.868464	-0.236562	-0.616780
C	4.826491	1.085692	-0.233186
C	3.460560	1.487051	-0.145998
H	5.689007	1.725678	-0.128341
C	3.142536	-2.080016	-0.810481
H	2.300763	-2.175382	-1.495772
H	3.967779	-2.642866	-1.246178
C	2.776157	-2.685865	0.535727
C	2.135590	-3.928374	2.969555
C	3.350818	-2.244317	1.730274
C	1.873517	-3.753230	0.575064
C	1.556973	-4.373466	1.781764
C	3.030940	-2.860699	2.939400
H	4.046144	-1.413007	1.719880
H	1.411341	-4.099069	-0.344057
H	0.853095	-5.198146	1.794649
H	3.482857	-2.504896	3.858628
H	1.887197	-4.406234	3.910382
C	6.058059	-1.050803	-0.917559
C	8.399049	-2.503072	-1.494322
C	7.138358	-1.061062	-0.021157
C	6.178911	-1.776109	-2.114936
C	7.334804	-2.499884	-2.395328
C	8.298165	-1.775530	-0.309384
H	7.058210	-0.511709	0.909740
H	5.375853	-1.749900	-2.842627
H	7.409116	-3.050688	-3.326226
H	9.120183	-1.771384	0.397584
H	9.299404	-3.064566	-1.715598
C	3.004456	2.847621	0.164599
C	2.207710	5.487691	0.759751
C	1.853438	3.407814	-0.420464
C	3.748380	3.651089	1.049475
C	3.363492	4.948952	1.345878

C	1.455817	4.702922	-0.129104
H	1.272940	2.825508	-1.123306
H	4.633436	3.243174	1.522383
H	3.947910	5.547373	2.033771
H	0.569949	5.117141	-0.594573
C	1.802316	6.822470	1.061619
N	1.473032	7.903968	1.306448

Zero-point correction =	0.638258 (Hartree/particle)
Sum of electronic and zero-point Energies	-1918.622731 a.u., -1203935.7637025 kcal/mol
Sum of electronic and thermal Energies	-1918.584536 a.u., 1203911.79634000 kcal/mol
Sum of electronic and thermal Enthalpies	-1918.583592 a.u., 1203911.20398 kcal/mol
Sum of electronic and thermal Free Energie	-1918.701087 a.u., 1203984.9320925 kcal/mol

PBEh1PBE 6-311G TD = (NStates = 10, root = 1) SCRF = (Solvent = Dichloromethane)**

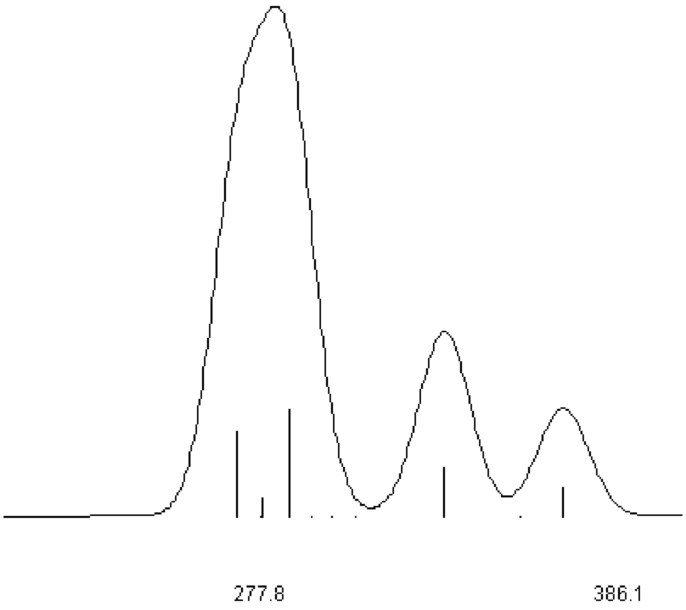

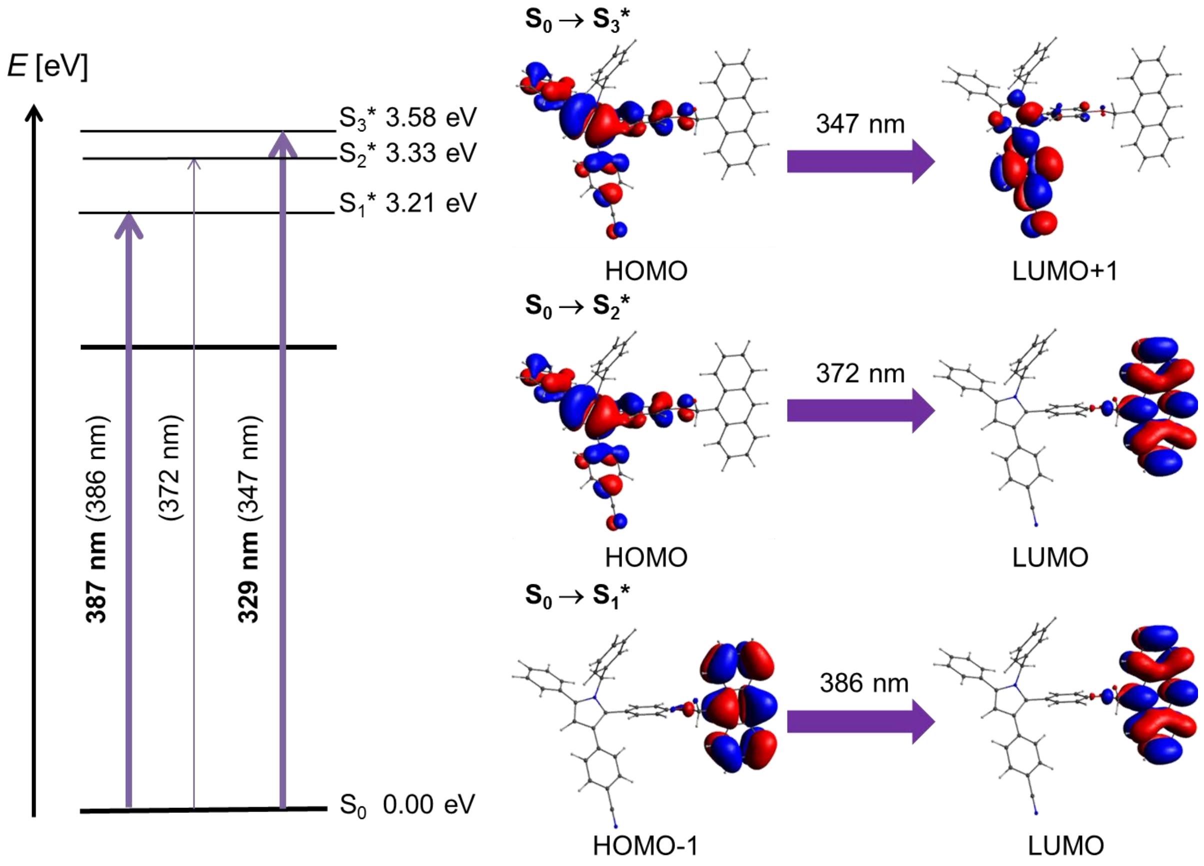

Figure S12. Jablonski diagram of compound **8** with experimental (bold) and calculated (in parentheses) absorption bands and assignment of the FMO-transitions ($E(S_0)$ = 0 eV; PBEh1PBE 6-311G** IEFPCM CH_2Cl_2, isosurface value at 0.03 a.u.).

3.3. Pyrrole-dansyl bichromophore 9

B3LYP 6-311G SCRF (Solvent = Dichloromethane) optimized S_0 ground state xyz-coordinates**

H	-5.179858	2.476910	1.841047
C	-5.707672	1.676663	1.340508
C	-5.121187	1.025456	0.278022
C	-7.649128	0.271083	1.124167
C	-5.763882	-0.040384	-0.429369
C	-7.002278	1.309722	1.749120
C	-7.047426	-0.453531	0.064893
C	-5.230493	-0.676841	-1.579053
H	-7.483372	1.855989	2.550651
H	-8.654891	0.003793	1.418813
C	-5.944885	-1.668614	-2.204621
H	-4.280399	-0.362606	-1.982533
H	-5.539574	-2.138223	-3.093749
C	-7.181379	-2.117290	-1.700949
H	-7.686577	-2.930569	-2.204601
C	-7.731330	-1.555698	-0.563355
N	-8.958061	-1.993236	-0.011860
C	-8.854289	-2.703811	1.271094
H	-8.164925	-2.195132	1.942393
H	-8.499681	-3.736938	1.132705
H	-9.837185	-2.737700	1.746777
C	-9.880756	-2.668473	-0.919107
H	-10.856940	-2.733663	-0.433644
H	-9.568098	-3.692325	-1.179468
H	-9.991868	-2.091612	-1.838034
S	-3.505019	1.630743	-0.185592
O	-3.093616	2.699932	0.715171
O	-3.393778	1.855795	-1.623164
O	-2.633894	0.257717	0.196375
C	-1.239251	0.295287	0.030073
C	1.534653	0.237300	-0.247570
C	-0.440926	0.761426	1.068797
C	-0.684007	-0.201352	-1.143580
C	0.700423	-0.224764	-1.278013

C	0.941741	0.727082	0.925585
H	-0.900152	1.133951	1.975051
H	-1.327514	-0.557851	-1.937231
H	1.137144	-0.587367	-2.201100
H	1.569978	1.078551	1.734579
C	3.003593	0.237760	-0.398105
N	3.731112	-0.918774	-0.641895
C	5.084024	-0.603130	-0.702134
C	5.205814	0.753059	-0.498198
C	3.902471	1.301439	-0.317069
H	6.128033	1.308876	-0.569340
C	3.214261	-2.277526	-0.455723
H	2.138931	-2.245166	-0.631384
H	3.635297	-2.923412	-1.224893
C	3.486140	-2.877193	0.915433
C	3.879790	-4.066098	3.427118
C	3.709522	-2.088218	2.045964
C	3.468456	-4.268908	1.056001
C	3.660202	-4.860451	2.302139
C	3.906296	-2.679569	3.293804
H	3.738428	-1.008808	1.955427
H	3.306577	-4.894658	0.183955
H	3.645781	-5.940795	2.392967
H	4.081593	-2.053622	4.161638
H	4.034116	-4.524510	4.397247
C	6.151910	-1.575676	-0.992150
C	8.264684	-3.341045	-1.567778
C	7.303310	-1.604195	-0.190601
C	6.084321	-2.442069	-2.095182
C	7.127779	-3.320145	-2.375042
C	8.350765	-2.475197	-0.478666
H	7.366167	-0.946222	0.668270
H	5.224783	-2.407135	-2.754746
H	7.058384	-3.978936	-3.233326
H	9.230993	-2.483604	0.154254
H	9.077530	-4.023356	-1.788400
C	3.611535	2.731736	-0.148213
C	3.135945	5.492627	0.167900

C	2.472438	3.339527	-0.706855
C	4.510120	3.547417	0.564563
C	4.283236	4.905343	0.723154
C	2.231481	4.695618	-0.551021
H	1.775793	2.746387	-1.284106
H	5.391183	3.104101	1.012434
H	4.984774	5.514094	1.280005
H	1.350509	5.145071	-0.992171
C	2.893951	6.890293	0.329444
N	2.699212	8.022736	0.460955

Zero-point correction =	0.644934 (Hartree/particle)
Sum of electronic and zero-point Energies =	-2408.266049 a.u., -1511186.9457 kcal/mol
Sum of electronic and thermal Energies =	-2408.224248a.u., 1511160.71562 kcal/mol
Sum of electronic and thermal Enthalpies =	-2408.223304a.u., 1511160.12326 Kcal/mol
Sum of electronic and thermal Free Energies =	-2408.349114a.u., 1511239.06904 kcal/mol

PBEh1PBE 6-311G TD = (NStates = 10, root = 1) SCRF = (Solvent = Dichloromethane)**

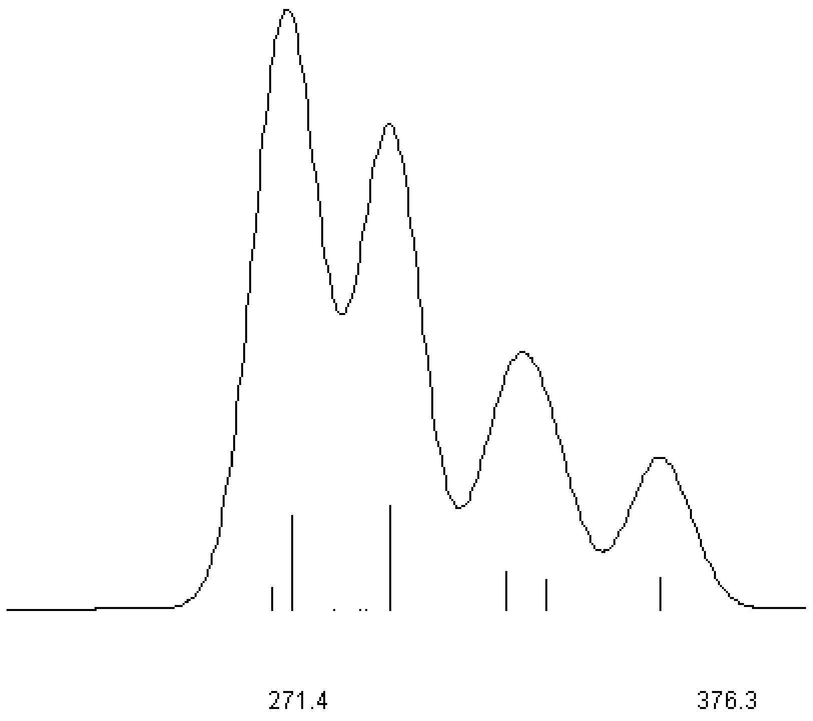

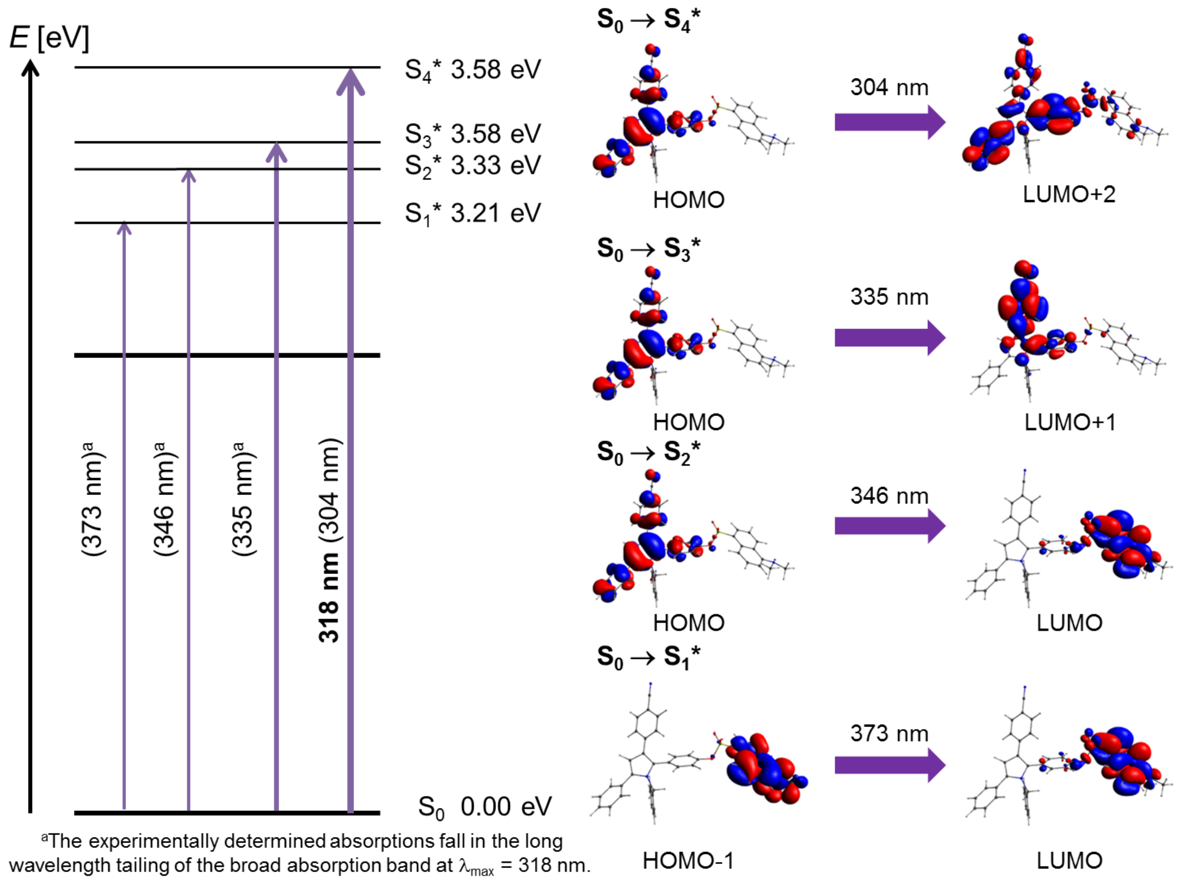

Figure S13. Jablonski diagram of compound **9** with experimental (bold) and calculated (in parentheses) absorption bands and assignment of the FMO-transitions ($E(S_0)$ = 0 eV; PBEh1PBE 6-311G** IEFPCM CH_2Cl_2, isosurface value at 0.03 a.u.).

3.4. Pyrrole-Nile red bichromophore 10

B3LYP 6-311G SCRF (Solvent = Dichloromethane) optimized S_0 ground state xyz-coordinates**

C	3.983510	-0.876722	-0.902377
C	5.703602	-2.455767	0.659094
C	3.495484	-2.029666	-0.263126
C	5.322948	-0.517353	-0.755072
C	6.188618	-1.293422	0.017961
C	4.358214	-2.799369	0.501837
H	2.460325	-2.327323	-0.354615
H	5.689474	0.371481	-1.250597
H	3.998638	-3.690806	1.000726
C	6.587354	-3.311941	1.491371
C	7.597552	-0.914753	0.171605
C	8.432089	-1.779683	0.992957
C	7.972563	-2.897055	1.611603
H	8.637807	-3.504906	2.211891
O	6.162254	-4.327980	2.053035
O	9.748231	-1.448146	1.148728
O	3.243022	-0.051288	-1.691905
C	1.848697	-0.311189	-1.912440
H	1.684629	-1.378753	-2.087136
H	1.621626	0.214525	-2.840896
C	10.245707	-0.325497	0.543408
C	11.281997	1.927786	-0.676152
C	11.580205	-0.034704	0.729013
C	9.386185	0.473107	-0.238596
C	9.954338	1.611079	-0.843474
C	12.144221	1.119950	0.129813
H	12.161769	-0.711279	1.336169
H	9.312879	2.234945	-1.454567
H	11.667141	2.805606	-1.171874
N	8.067870	0.156394	-0.410165
N	13.456292	1.452932	0.325481
C	14.341666	0.607361	1.136667
H	13.803866	0.286648	2.032362
H	15.156287	1.243172	1.485855
C	14.044588	2.647355	-0.295424

H	14.900769	2.933271	0.317273
H	13.333412	3.473231	-0.222929
C	14.494860	2.459438	-1.748802
H	13.665035	2.156357	-2.390798
H	15.277455	1.701856	-1.824860
H	14.896060	3.400766	-2.134343
C	14.919074	-0.607743	0.400956
H	14.130874	-1.261599	0.022002
H	15.542183	-1.189866	1.085383
H	15.539375	-0.299359	-0.442888
C	0.983230	0.222654	-0.772296
H	1.237854	-0.274110	0.167233
H	1.185383	1.289431	-0.645222
C	-0.499303	0.022602	-1.049615
H	-0.740132	-1.041843	-1.153676
H	-0.798531	0.535569	-1.971027
O	-1.211522	0.572950	0.064702
C	-2.571875	0.518956	0.071086
C	-5.384731	0.510804	0.284804
C	-3.351957	-0.041281	-0.944631
C	-3.200173	1.075510	1.193598
C	-4.581581	1.067650	1.295835
C	-4.742306	-0.037516	-0.830077
H	-2.897186	-0.473805	-1.825201
H	-2.583839	1.503903	1.974877
H	-5.052482	1.497056	2.172385
H	-5.331270	-0.462128	-1.634424
C	-6.858694	0.542660	0.416996
N	-7.616439	-0.603111	0.600935
C	-8.948302	-0.253239	0.792364
C	-9.026897	1.119591	0.736874
C	-7.719134	1.638742	0.503258
H	-9.935538	1.690267	0.851064
C	-7.108118	-1.966501	0.484823
H	-7.595608	-2.584876	1.238128
H	-6.047586	-1.941657	0.739842
C	-7.289639	-2.611270	-0.882518
C	-7.561829	-3.893637	-3.365013

C	-6.872723	-3.937188	-1.050451
C	-7.846177	-1.937832	-1.970438
C	-7.980989	-2.575868	-3.205116
C	-7.006046	-4.574027	-2.280549
H	-6.440781	-4.474307	-0.211636
H	-8.179766	-0.913042	-1.858984
H	-8.416691	-2.038720	-4.040171
H	-6.677860	-5.601293	-2.393147
H	-7.667870	-4.388680	-4.323525
C	-7.388631	3.064015	0.380151
C	-6.835194	5.821266	0.136259
C	-6.350859	3.526716	-0.451136
C	-8.143953	4.024548	1.080049
C	-7.878829	5.379773	0.964161
C	-6.072721	4.878780	-0.571911
H	-5.764954	2.817885	-1.020479
H	-8.940986	3.699875	1.737486
H	-8.469882	6.099783	1.516598
H	-5.272863	5.212932	-1.221214
C	-6.553872	7.214968	0.012660
N	-6.325791	8.344550	-0.087839
C	-10.043716	-1.214745	1.029974
C	-12.203924	-2.952289	1.507241
C	-11.181746	-1.188950	0.210056
C	-10.014590	-2.120210	2.102480
C	-11.081245	-2.985056	2.333540
C	-12.252972	-2.046579	0.448987
H	-11.217352	-0.496634	-0.623014
H	-9.166419	-2.127800	2.777563
H	-11.040757	-3.675170	3.168738
H	-13.122740	-2.012019	-0.197397
H	-13.035024	-3.623759	1.690065

Zero-point correction=	0.842924 (Hartree/particle)
Sum of electronic and zero-point Energies=	-2565.832369a.u., -1610059.8115475 kcal/mol
Sum of electronic and thermal Energies=	-2565.779888a.u., 1610026.87972 kcal/mol
Sum of electronic and thermal Enthalpies=	-2565.778944a.u., 1610026.2873600002 kcal/mol
Sum of electronic and thermal Free Energies=	-2565.931021a.u., 1610121.7156775 kcal/mol

PBEh1PBE 6-311G TD = (NStates = 10, root = 1) SCRF = (Solvent = Dichloromethane)**

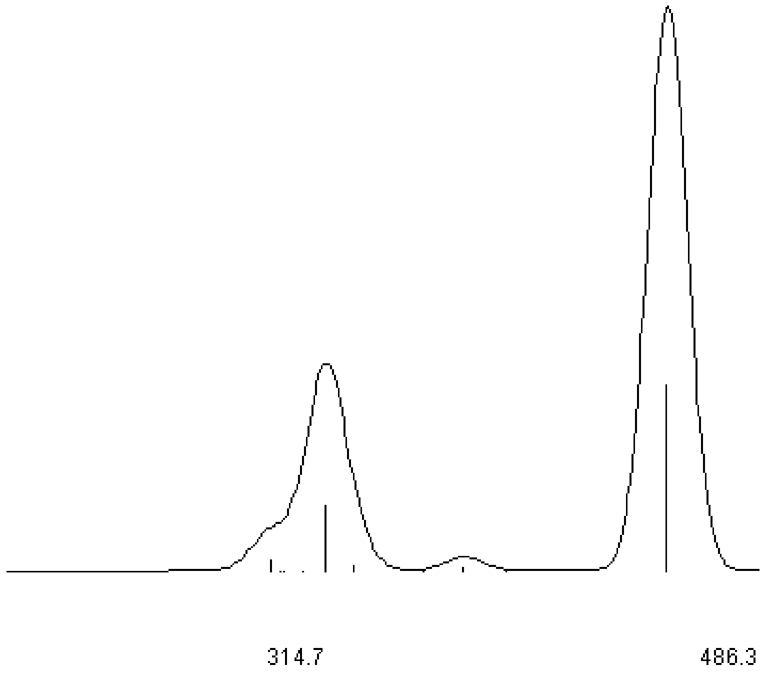

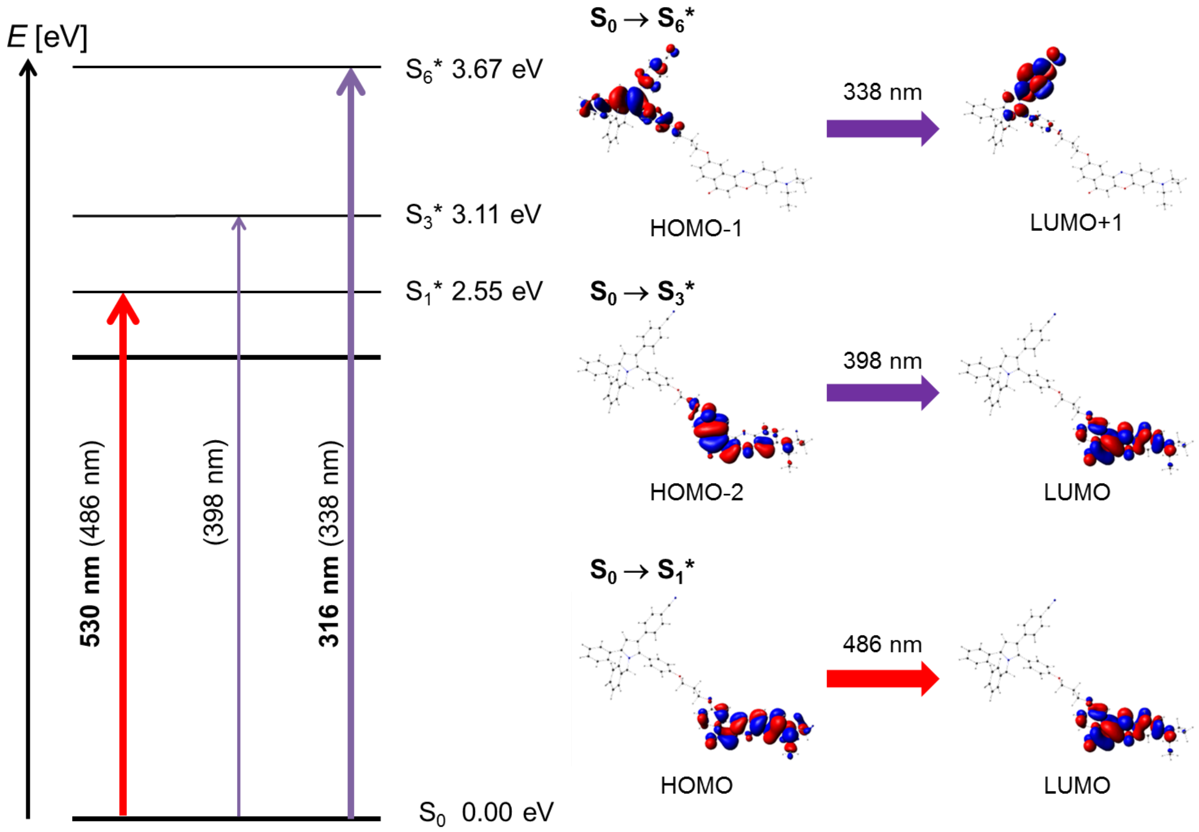

Figure S14. Jablonski diagram of compound **10** with experimental (bold) and calculated (in parentheses) absorption bands and assignment of the FMO-transitions ($E(S_0)$ = 0 eV; PBEh1PBE 6-311G** IEFPCM CH_2Cl_2, isosurface value at 0.03 a.u.) (Nile red localized intense absorption in red and pyrrole localized intense absorption in violet).

3.5. Pyrrole-3-cyano-5,5-dimethylfuran-2(5*H*)-ylidene)malononitrile-styryl bichromophore 11

B3LYP 6-311G SCRF (Solvent = Dichloromethane) optimized S_0 ground state xyz-coordinates**

C	9.045899	-1.594897	-0.812358
O	10.410885	-2.091595	-0.543138
C	11.070607	-1.259095	0.260733
C	10.233469	-0.145059	0.603122
C	8.998252	-0.298657	-0.017135
C	12.372743	-1.573878	0.619727
C	8.097386	-2.676513	-0.291263
H	8.325467	-3.621299	-0.787319
H	7.059275	-2.417744	-0.500058
H	8.214360	-2.805314	0.785749
C	8.964175	-1.394540	-2.327123
H	9.183980	-2.339969	-2.825801
H	9.685482	-0.645095	-2.656070
H	7.966712	-1.069981	-2.623707
C	13.157866	-0.760071	1.472341
N	13.835027	-0.126497	2.165742
C	12.967667	-2.763294	0.124556
N	13.466417	-3.729951	-0.271627
C	10.575250	0.948342	1.430683
N	10.794190	1.868556	2.096878
C	7.922125	0.608516	0.095798
H	8.116047	1.474170	0.720047
C	6.692334	0.487266	-0.501150
H	6.501439	-0.380559	-1.123136
C	5.591075	1.394487	-0.403195
C	3.319420	3.130977	-0.288747
C	4.392173	1.101612	-1.095516
C	5.611356	2.590604	0.354192
C	4.522927	3.427995	0.414540
C	3.292476	1.927496	-1.045435
H	4.334815	0.191299	-1.682814
H	6.501003	2.870344	0.905180
H	4.595823	4.330157	1.004031
H	2.403750	1.636825	-1.586601
N	2.251129	3.977926	-0.228414

C	1.028404	3.740600	-0.990031
H	1.260565	3.268446	-1.944812
H	0.572949	4.704482	-1.220316
C	2.293042	5.170010	0.619877
H	3.054095	5.878088	0.277458
H	2.497272	4.912072	1.662314
H	1.325023	5.663920	0.576822
C	0.020494	2.882936	-0.222027
H	0.443711	1.899661	0.008899
H	-0.260214	3.371426	0.717401
O	-1.116326	2.750986	-1.075135
C	-2.191561	2.031722	-0.642978
C	-4.512050	0.593225	0.056768
C	-3.272476	1.955281	-1.530348
C	-2.269833	1.389763	0.595779
C	-3.424326	0.684066	0.933188
C	-4.410374	1.244396	-1.184288
H	-3.195780	2.455603	-2.488165
H	-1.454201	1.437671	1.304266
H	-3.480863	0.210961	1.907173
H	-5.235597	1.189079	-1.884179
C	-5.742113	-0.134605	0.429103
N	-5.747077	-1.491674	0.718635
C	-7.048542	-1.898165	0.995741
C	-7.862923	-0.794802	0.878072
C	-7.053904	0.329114	0.535224
H	-8.916267	-0.776571	1.112124
C	-4.650290	-2.407823	0.398576
H	-3.727488	-1.827629	0.415985
H	-4.566838	-3.151184	1.190292
C	-7.423919	-3.264018	1.400072
C	-8.246032	-5.829976	2.209898
C	-8.513602	-3.898003	0.783673
C	-6.760310	-3.937562	2.438472
C	-7.163730	-5.210067	2.833654
C	-8.922963	-5.166034	1.188037
H	-9.030657	-3.393644	-0.024309
H	-5.944012	-3.450810	2.959862

H	-6.640539	-5.712050	3.639763
H	-9.765915	-5.639450	0.697300
H	-8.560713	-6.819702	2.520315
C	-7.547525	1.704697	0.393194
C	-8.572669	4.321457	0.146159
C	-6.784022	2.822792	0.777574
C	-8.841891	1.935555	-0.110325
C	-9.352286	3.218002	-0.233737
C	-7.281428	4.110416	0.655172
H	-5.795145	2.678888	1.191652
H	-9.448954	1.094745	-0.423154
H	-10.348692	3.372568	-0.628904
H	-6.679619	4.956941	0.962063
C	-4.781444	-3.110361	-0.944285
C	-4.888029	-4.421466	-3.423801
C	-5.554932	-2.594460	-1.986353
C	-4.066544	-4.294095	-1.156298
C	-4.115515	-4.944147	-2.386945
C	-5.608559	-3.247035	-3.218026
H	-6.124923	-1.685044	-1.837577
H	-3.469774	-4.712021	-0.351570
H	-3.557347	-5.862108	-2.533420
H	-6.216418	-2.835795	-4.016223
H	-4.931797	-4.928566	-4.380912
C	-9.088843	5.646151	0.019985
N	-9.506187	6.720056	-0.082465

Zero-point correction =	0.781805 (Hartree/particle)
Sum of electronic and zero-point Energies =	-2446.235861 a.u., -1535013.0027775 kcal/mol
Sum of electronic and thermal Energies =	-2446.183037 a.u., 1534979.8557175 kcal/mol
Sum of electronic and thermal Enthalpies =	-2446.182093 a.u., 1534979.2633574998 kcal/mol
Sum of electronic and thermal Free Energies =	-2446.334704 a.u., 1535075.02676 kcal/mol

PBEh1PBE 6-311G TD = (NStates = 10, root = 1) SCRF = (Solvent = Dichloromethane)**

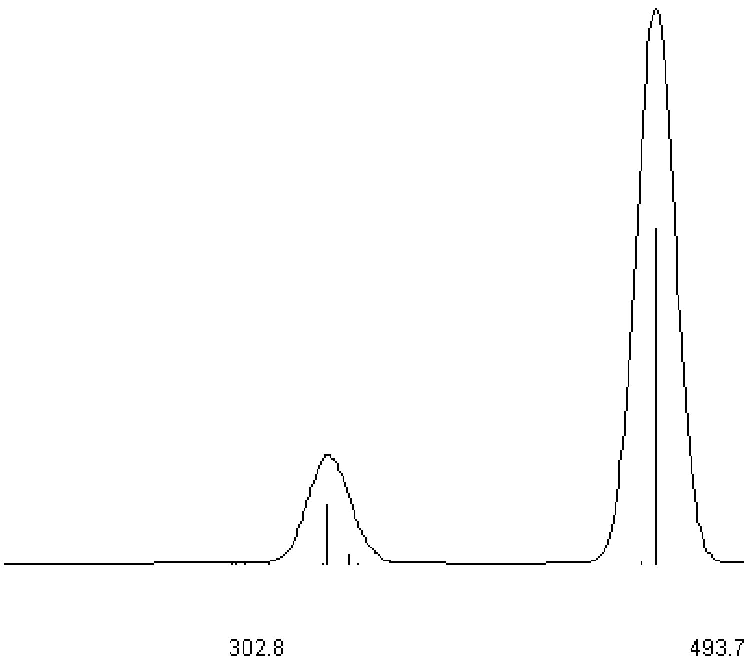

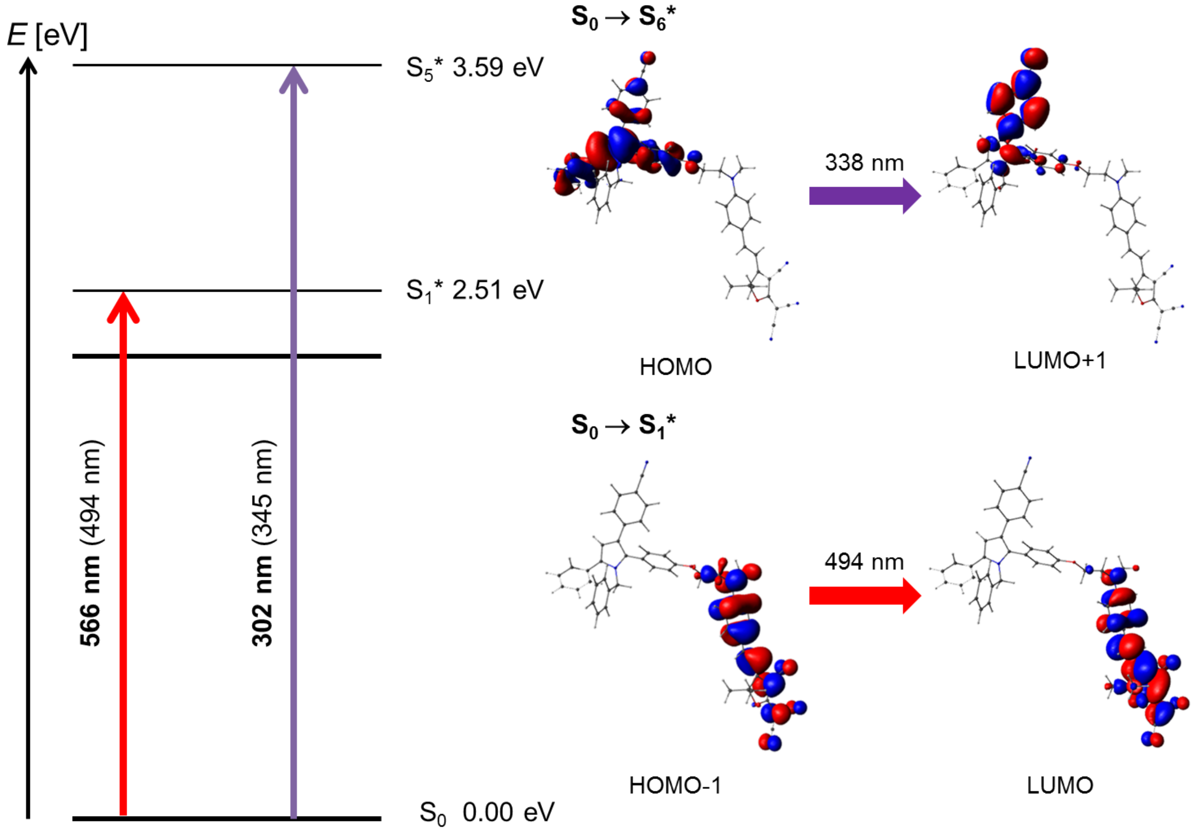

Figure S15. Jablonski diagram of compound **11** with experimental (bold) and calculated (in parentheses) absorption bands and assignment of the FMO-transitions ($E(S_0)$ = 0 eV; PBEh1PBE 6-311G** IEFPCM CH_2Cl_2, isosurface value at 0.03 a.u.) (3-cyano-5,5-dimethylfuran-2(5*H*)-ylidene)malononitrile-styrene localized intense absorption in red and pyrrole localized intense absorption in violet).

3.6. Pyrrole-quinoxalinyl-styryl bichromophore 12

B3LYP 6-311G SCRF (Solvent = Dichloromethane) optimized S_0 ground state xyz-coordinates**

H	-9.256776	-3.587999	-1.810470
C	-10.113475	-3.281682	-1.222323
C	-12.276285	-2.411302	0.320539
C	-10.028008	-2.049000	-0.547214
C	-11.250026	-4.063239	-1.131137
C	-12.331386	-3.621640	-0.356413
C	-11.125131	-1.612289	0.233280
H	-11.305845	-5.010302	-1.654037
H	-13.225989	-4.228480	-0.280106
H	-13.123311	-2.092756	0.911410
N	-8.880972	-1.308096	-0.666265
N	-11.009043	-0.390856	0.888462
C	-9.868514	0.398799	0.799661
C	-12.119644	0.101626	1.709913
H	-13.013597	0.230213	1.097056
H	-11.822445	1.057640	2.126902
H	-12.331344	-0.601683	2.517228
C	-8.770820	-0.157265	-0.049019
C	-7.517706	0.549330	-0.234276
H	-6.844287	-0.007571	-0.878661
C	-7.128946	1.742734	0.277480
H	-7.825095	2.272035	0.915268
C	-5.851599	2.399003	0.056160
C	-3.373156	3.796174	-0.301442
C	-4.810208	1.870320	-0.733648
C	-5.605782	3.644735	0.661956
C	-4.412828	4.328546	0.497255
C	-3.612600	2.535471	-0.908148
H	-4.933847	0.909717	-1.220130
H	-6.379541	4.089384	1.279712
H	-4.290629	5.284660	0.985994
H	-2.850326	2.065817	-1.514195
N	-2.189132	4.479150	-0.473919
C	-1.144970	4.003400	-1.369518
H	-0.602053	4.864833	-1.762690

H	-1.581516	3.492886	-2.229481
C	-1.958448	5.735545	0.230310
H	-2.058492	5.614911	1.313412
H	-2.654083	6.518194	-0.094532
H	-0.945578	6.075605	0.022965
O	-9.794201	1.474185	1.387981
C	-0.152387	3.070828	-0.671681
H	-0.660584	2.181837	-0.284088
H	0.333477	3.587125	0.163453
O	0.814651	2.701975	-1.658156
C	1.834719	1.868718	-1.310043
C	4.017791	0.160120	-0.797517
C	2.742066	1.553385	-2.330208
C	2.018515	1.328234	-0.034336
C	3.103596	0.487427	0.208633
C	3.812114	0.710130	-2.075715
H	2.582441	1.972511	-3.316385
H	1.335375	1.555528	0.772596
H	3.239601	0.080418	1.203558
H	4.490720	0.458524	-2.883157
C	5.142042	-0.759633	-0.529608
N	6.467286	-0.372381	-0.665486
C	7.295643	-1.431095	-0.305776
C	6.488493	-2.485317	0.057152
C	5.127312	-2.084373	-0.091834
H	6.839970	-3.474505	0.307293
C	6.909362	1.017902	-0.779482
H	6.133281	1.566689	-1.313330
H	7.800963	1.051665	-1.404250
C	7.195652	1.708722	0.546027
C	7.713686	3.096870	2.930258
C	7.891345	2.923106	0.527952
C	6.767166	1.196238	1.771625
C	7.025758	1.886403	2.956785
C	8.146718	3.614223	1.709073
H	8.237070	3.329686	-0.417464
H	6.234695	0.253456	1.807452
H	6.688617	1.473576	3.901073

H	8.688419	4.552966	1.677093
H	7.914944	3.631471	3.851582
C	8.766542	-1.399980	-0.381466
C	11.576231	-1.462979	-0.517447
C	9.440019	-0.986715	-1.542622
C	9.529272	-1.854844	0.705176
C	10.919589	-1.890263	0.635591
C	10.830566	-1.011765	-1.606078
H	8.871965	-0.673539	-2.411182
H	9.023827	-2.170256	1.610518
H	11.490449	-2.243427	1.487021
H	11.331496	-0.691829	-2.512854
H	12.658723	-1.484707	-0.569309
C	3.967950	-2.958197	0.128082
C	1.794963	-4.704820	0.561278
C	4.021771	-3.956303	1.119618
C	2.796156	-2.865134	-0.645973
C	1.724652	-3.718230	-0.435095
C	2.958766	-4.818275	1.337331
H	4.906934	-4.045518	1.737578
H	2.731348	-2.125249	-1.432353
H	0.834227	-3.633464	-1.045722
H	3.020138	-5.575608	2.109076
C	0.694960	-5.587626	0.779879
N	-0.196128	-6.303512	0.957147

Zero-point correction=	0.787995 (Hartree/Particle)
Sum of electronic and zero-point Energies=	-2354.021558 a.u., -1477148.527645 kcal/mol
Sum of electronic and thermal Energies=	-2353.972684 a.u., 1477117.85921 kcal/mol
Sum of electronic and thermal Enthalpies=	-2353.971740 a.u., 1477117.26685 kcal/mol
Sum of electronic and thermal Free Energies=	-2354.115952 a.u., 1477207.75988 kcal/mol

PBEh1PBE 6-311G TD = (NStates = 10, root = 1) SCRF = (Solvent = Dichloromethane)**

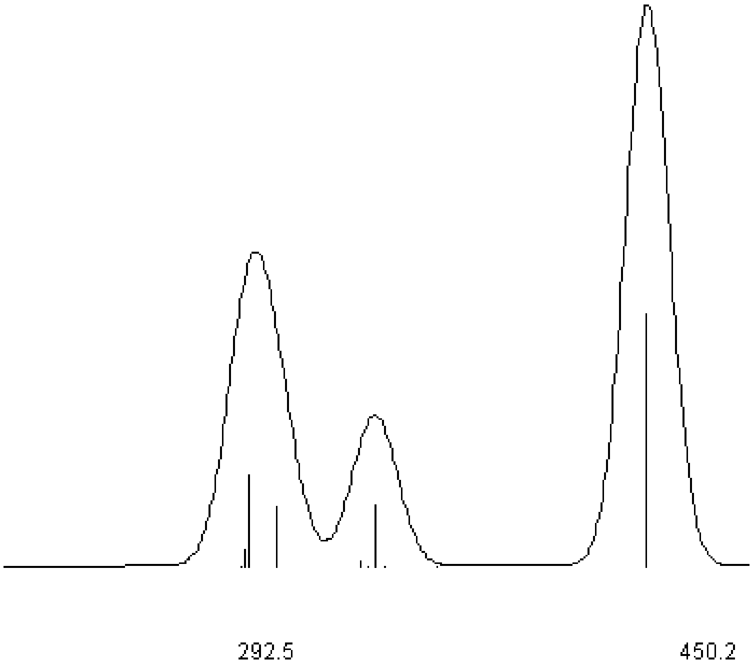

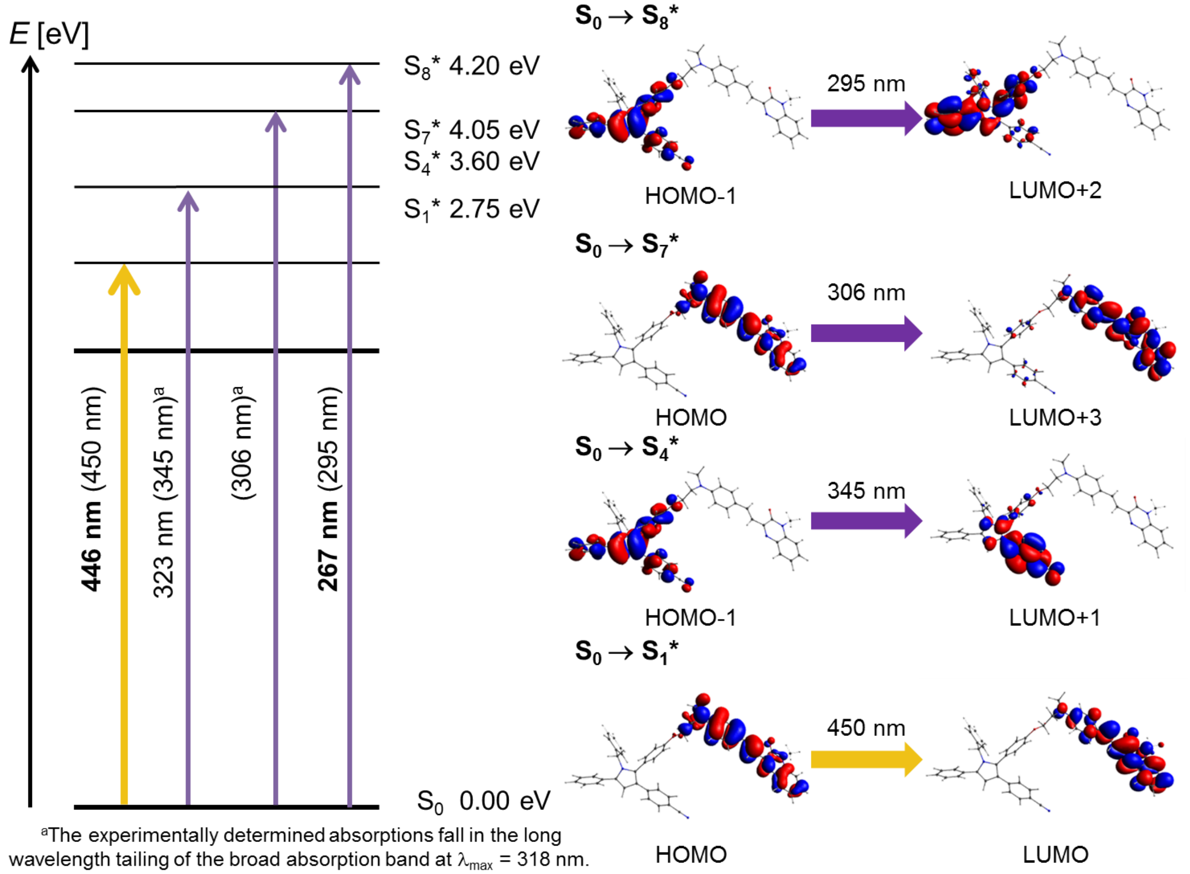

Figure S16. Jablonski diagram of compound **12** with experimental (bold) and calculated (in parentheses) absorption bands and assignment of the FMO-transitions ($E(S_0)$ = 0 eV; PBEh1PBE 6-311G** IEFPCM CH_2Cl_2, isosurface value at 0.03 a.u.) (quinoxalinyl-styrene localized intense absorption in yellow).

3.7. Assignment of the TD-DFT calculated transitions

Table S1. Assignment of the S_1-S_{10} states of the chromophores of **2** and **8-12** (λ_{max} [nm], oscillator strength *f*, calculated with $PBE_1PBE/6\text{-}311G^{**}/CH_2Cl_2$)

	$\lambda_{max,exp}$ [nm] (ε) [L mol^{-1} cm^{-1}]	State	$\lambda_{max,calcd}$ [nm] ([eV])	*f*	Contributions of the dominant orbital transitions
**2**	316 (20200)	**S_1**	**347.2 (3.5713)**	**0.3151**	**HOMO → LUMO (98.7%)**
		**S_2**	**294.1 (4.2152)**	**0.6305**	**HOMO → LUMO+1 (97.0%)**
	276 (30600)	S_3	287.5 (4.3123)	0.0292	HOMO → LUMO+2 (93.0%)
		**S_4**	**278.5 (4.4524)**	**0.5327**	**HOMO-2 → LUMO (6.5%), HOMO-1 → LUMO (84.6%), HOMO → LUMO+3 (4.3%)**
		S_5	272.3 (4.5534)	0.0492	HOMO-2 → LUMO (45.2%), HOMO → LUMO+3 (47.7%)
		S_6	270.7 (4.5802)	0.0085	HOMO-2 → LUMO (42.5%), HOMO-1 → LUMO (9.3%), HOMO → LUMO+3 (37.2%), HOMO → LUMO+4 (4.1%)
		S_7	268.5 (4.6177)	0.0069	HOMO-8 → LUMO (3.5%), HOMO → LUMO+4 (81.2%), HOMO → LUMO+6 (4.4%)
		S_8	258.9 (4.7894)	0.0027	HOMO → LUMO+5 (81.2%), HOMO → LUMO+6 (12.1%)
		S_9	254.7 (4.8679)	0.0127	HOMO → LUMO+3 (2.7%), HOMO → LUMO+4 (5.7%), HOMO → LUMO+5 (14.1%), HOMO -> LUMO+6 (71.0%)
		S_{10}	248.0 (5.0003)	0.0293	HOMO-8 → LUMO (6.7%), HOMO-6 → LUMO (3.4%), HOMO-2 → LUMO+2 (2.9%), HOMO-1 → LUMO+2 (6.3%), HOMO → LUMO+7 (66.0%)
**8**	387 (14200)	S_1	**386.1 (3.2110)**	**0.1701**	**HOMO-1 → LUMO (98.2%)**
		S_2	372.4 (3.3292)	0.0001	HOMO → LUMO (97.3%)
	367 (18100)	S_3	**346.7 (3.5764)**	**0.2919**	**HOMO → LUMO+1 (98.4%)**
	329 (29600)	S_4	317.3 (3.9081)	0.0052	HOMO-4 → LUMO (49.1%); HOMO-1 → LUMO+3 (37.7%)
		S_5	309.5 (4.0061)	0.0004	HOMO-1 → LUMO+1 (99.0%)
		S_6	302.3 (4.1009)	0.0004	HOMO-3 → LUMO (30.3%); HOMO-2 → LUMO (59.0%)
	315.5 (29800)	S_7	**295.1 (4.2012)**	**0.6313**	**HOMO → LUMO+2 (95.2%)**
		S_8	**286.2 (4.3327)**	**0.1106**	**HOMO → LUMO+3 (8.5%); HOMO → LUMO+4 (83.3%)**
		S_9	285.5 (4.3423)	0.0001	HOMO-3 → LUMO (64.2%); HOMO-2 → LUMO (35.3%)
	258 (192800)	S_{10}	**277.8 (4.4638)**	**0.5081**	**HOMO-3 → LUMO+1 (11.1%); HOMO-2 → LUMO+1 (79.6%); HOMO → LUMO+5 (3.4%)**
**9**	318 (32200)	S_1	**376.3 (3.295)**	**0.1694**	**HOMO-1 → LUMO (96.3%)**
		S_2	**345.6 (3.5873)**	**0.1500**	**HOMO → LUMO (65.7%) HOMO → LUMO+1 (30.6%)**
		S_3	**335.1 (3.6997)**	**0.1957**	**HOMO → LUMO (31.1%); HOMO → LUMO+1 (67.4%)**
		S_4	**303.6 (4.0841)**	**0.5256**	**HOMO → LUMO+2 (95.4%)**
		S_5	297.1 (4.1729)	0.0071	HOMO-8 → LUMO (10.7%); HOMO-1 → LUMO+1 (46.7%); HOMO-1 → LUMO+2 (4.0%); HOMO-1 → LUMO+3 (36.2%)
		S_6	295.2 (4.1998)	0.0094	HOMO-8 → LUMO (10.0%); HOMO-1 → LUMO+1 (50.3%); HOMO-1 → LUMO+3 (36.8%)
		S_7	288.5 (4.2976)	0.0049	HOMO → LUMO+4 (93.3%)
	266 (54400)	S_8	**276.9 (4.4776)**	**0.4759**	**HOMO-2 → LUMO (41.5%); HOMO-2 → LUMO+1 (52.0%)**

		S_9	**271.9 (4.5599)**	**0.1007**	**HOMO-4 → LUMO (37.9%); HOMO-2 → LUMO (17.9%); HOMO-2 → LUMO+1 (14.9%); HOMO-1 → LUMO+2 (15.6%)**
		S_{10}	**271.4 (4.5678)**	**0.1183**	**HOMO-4 → LUMO (20.5%); HOMO-2 → LUMO (37.6%); HOMO-2 → LUMO+1 (20.3%); HOMO-1 → LUMO+2 (10.5%)**
	529.5 (10500)	S_1	**486.3 (2.5494)**	**0.9954**	**HOMO -> LUMO (98.4%)**
		S_2	416.8 (2.9746)	0.0000	HOMO-1 -> LUMO (99.8%)
		S_3	398.2 (3.1135)	0.0239	HOMO-2 -> LUMO (96.6%)
		S_4	381.0 (3.2539)	0.0000	HOMO-7 -> LUMO (93.2%); HOMO-7 -> LUMO+2 (4.9%)
		S_5	350.6 (3.5360)	0.0364	HOMO-5 -> LUMO (87.4%); HOMO -> LUMO+2 (6.3%)
**10**	316 (6000)	S_6	**338.2 (3.6657)**	**0.3500**	**HOMO-1 -> LUMO+1 (98.5%)**
		S_7	328.9 (3.7699)	0.0000	HOMO-4 -> LUMO (15.8%); HOMO-3 -> LUMO (83.9%)
		S_8	320.1 (3.8729)	0.0012	HOMO-15 -> LUMO (97.3%)
		S_9	319.1 (3.8855)	0.0000	HOMO-4 -> LUMO (83.9%); HOMO-3 -> LUMO (16.0%)
	294 (7200)<br>267.5 (16200)	S_{10}	314.7 (3.9401)	0.0682	HOMO-10 -> LUMO (11.8%)
	566 (51900)	S_1	**493.7 (2.5114)**	**1.8138**	**HOMO-1 -> LUMO (98.0%)**
		S_2	487.1 (2.5455)	0.0181	HOMO -> LUMO (97.8%)
		S_3	359.8 (3.4463)	0.0008	HOMO-3 -> LUMO (8.8%); HOMO-2 -> LUMO (90.6%)
		S_4	355.3 (3.4895)	0.0584	HOMO-4 -> LUMO (92.4%); HOMO-3 -> LUMO (2.3%)
	302 (34700)	S_5	**345.2 (3.5916)**	**0.3254**	**HOMO -> LUMO+1 (98.4%)**
**11**		S_6	343.7 (3.6079)	0.0006	HOMO-4 -> LUMO (3.1%); HOMO-3 -> LUMO (88.2%); HOMO-2 -> LUMO (8.2%)
		S_7	319.5 (3.8809)	0.0000	HOMO-5 -> LUMO (99.4%)
		S_8	308.6 (4.0175)	0.0032	HOMO-12 -> LUMO (5.6%); HOMO-11 -> LUMO (84.4%); HOMO-1 -> LUMO+5 (6.7%)
		S_9	304.7 (4.0697)	0.0000	HOMO-6 -> LUMO (98.0%)
		S_{10}	302.8 (4.0946)	0.0003	HOMO-1 -> LUMO+1 (99.5%)
	446 (51700)	S_1	**450.2 (2.7537)**	**1.3556**	**HOMO -> LUMO (99.3%)**
		S_2	368.9 (3.3614)	0.0001	HOMO-1 -> LUMO (99.8%)
		S_3	348.6 (3.5565)	0.0024	HOMO -> LUMO+1 (99.2%)
		S_4	**344.7 (3.5967)**	**0.3340**	**HOMO-1 -> LUMO+1 (97.8%)**
		S_5	342.2 (3.6234)	0.0005	HOMO-7 -> LUMO (95.9%)
**12**		S_6	339.3 (3.6546)	0.0354	HOMO-5 -> LUMO (6.5%); HOMO-2 -> LUMO (89.7%)
		S_7	**306.3 (4.0483)**	**0.3206**	**HOMO-5 -> LUMO (29.3%); HOMO -> LUMO+3 (61.0%)**
	267 (54700)	S_8	**295.2 (4.2000)**	**0.4921**	**HOMO-5 -> LUMO (9.3%); HOMO-1 -> LUMO+2 (76.7%)**
		S_9	294.3 (4.2127)	0.0960	HOMO-5 -> LUMO (22.5%); HOMO-1 -> LUMO+2 (18.4%); HOMO -> LUMO+7 (12.1%); HOMO -> LUMO+8 (15.0%)
		S_{10}	292.5 (4.2390)	0.0063	HOMO-5 -> LUMO (3.7%); HOMO-4 -> LUMO (2.8%); HOMO-3 -> LUMO (89.0%)
